# Plasmodium falciparum and TNF-α Differentially Regulate Inflammatory and Barrier Integrity Pathways in Human Brain Endothelial Cells

**DOI:** 10.1128/mbio.01746-22

**Published:** 2022-08-29

**Authors:** Marisol Zuniga, Claudia Gomes, Ze Chen, Criseyda Martinez, Joseph Cooper Devlin, P’ng Loke, Ana Rodriguez

**Affiliations:** a Department of Microbiology, New York University School of Medicine, New York, New York, USA; b Department of Biochemistry and Molecular Pharmacology, New York University School of Medicine, New York, New York, USA; c Laboratory of Parasitic Diseases, National Institute of Allergy and Infectious Diseases, National Institutes of Health, Bethesda, Maryland, USA; Rutgers—New Jersey Medical School

**Keywords:** blood-brain barrier, *Plasmodium falciparum*, RNA-seq, barrier function, cerebral malaria, endothelial cells, inflammation, malaria, permeability, signaling pathways, tumor necrosis factor

## Abstract

Cerebral malaria is a severe complication of Plasmodium falciparum infection characterized by the loss of blood-brain barrier (BBB) integrity, which is associated with brain swelling and mortality in patients. P. falciparum-infected red blood cells and inflammatory cytokines, like tumor necrosis factor alpha (TNF-α), have been implicated in the development of cerebral malaria, but it is still unclear how they contribute to the loss of BBB integrity. Here, a combination of transcriptomic analysis and cellular assays detecting changes in barrier integrity and endothelial activation were used to distinguish between the effects of P. falciparum and TNF-α on a human brain microvascular endothelial cell (HBMEC) line and in primary human brain microvascular endothelial cells. We observed that while TNF-α induced high levels of endothelial activation, it only caused a small increase in HBMEC permeability. Conversely, P. falciparum-infected red blood cells (iRBCs) led to a strong increase in HBMEC permeability that was not mediated by cell death. Distinct transcriptomic profiles of TNF-α and P. falciparum in HBMECs confirm the differential effects of these stimuli, with the parasite preferentially inducing an endoplasmic reticulum stress response. Our results establish that there are fundamental differences in the responses induced by TNF-α and P. falciparum on brain endothelial cells and suggest that parasite-induced signaling is a major component driving the disruption of the BBB during cerebral malaria, proposing a potential target for much needed therapeutics.

## INTRODUCTION

Despite large efforts to reduce the global burden of malaria, over 600,000 deaths were reported in 2020, with children under the age of 5 accounting for most of those deaths (1). A major cause of death by malaria is cerebral malaria (CM), a severe neurological complication of Plasmodium falciparum infection with clinical features that include coma and seizures and which frequently leads to death (2). Mortality in children with CM is caused by brain swelling (3), which is thought to occur as a consequence of increased permeability of the blood-brain barrier (BBB) resulting in vasogenic edema (4). No specific therapies for CM are available; therefore, current treatments can only rely on classical antimalarial drugs to kill the parasite.

P. falciparum-infected red blood cells (iRBCs) bind to the endothelial lining of blood vessels through a variable *P. falciparum* protein called PfEMP1 and can cause microvascular obstruction. In particular, iRBC binding to endothelial protein C (EPCR) and intercellular adhesion molecule-1 (ICAM-1) receptors in brain microvascular endothelial cells is associated with CM (5). Binding of iRBCs to brain capillaries is thought to be a critical step for the disruption of the BBB since dense sequestration of iRBCs is found near hemorrhage sites in the brains of deceased CM patients (6). Densely packed capillaries impede blood flow and allow for the generation of microenvironments where P. falciparum-iRBC-derived factors can accumulate in high concentrations upon the rupture and release of the iRBC contents after the completion of the erythrocytic infection cycle. This is important due to mounting evidence suggesting that P. falciparum-iRBC-derived factors can disrupt brain endothelial integrity (7, 8).

Inflammation is an important aspect of malarial pathogenesis, as indicated by the correlation of inflammatory cytokine levels and disease severity (9, 10). Particularly, elevated levels of tumor necrosis factor alpha (TNF-α) have been implicated in CM pathogenesis due to its correlation with mortality in CM patients (11). TNF-α is a strong activator of endothelial cells, which results in increased levels of surface ICAM-1 (12, 13), facilitating increased sequestration of P. falciparum-iRBCs and contributing to CM pathogenesis (14, 15). TNF-α can also disrupt endothelial barrier integrity *in vitro* (16–18), but it is still uncertain whether it has a significant role in the breakdown of the BBB in the context of CM since the statistical relationship between TNF-α levels and brain swelling in CM patients is inconsistent (19, 20).

Inflammatory cytokines and inflammation in general have extensively been implicated in CM pathogenesis. As a result, the call for adjunctive therapies for CM has largely been focused on immunomodulatory strategies (21, 22). However, clinical trials to reduce circulating TNF-α by monoclonal antibodies or pentoxifylline did not improve survival in children with CM (23, 24). Novel therapies directed at the inhibition of P. falciparum sequestration and endothelial dysfunction, including BBB breakdown and endothelial activation (25) have been proposed, but successful endothelial-cell-targeted therapies have yet to be developed. Significant evidence implicates both TNF-α and P. falciparum in CM pathogenesis, but the cellular and molecular mechanisms underlying their effects are not clearly defined, hindering the development of effective targeted therapies. Specifically, their effect on the endothelium, a vital component of the BBB, remains ambiguous.

The aim of this study is to differentiate between the effects of TNF-α and P. falciparum on human brain endothelial cells (HBMECs) in order to gain an understanding of their role in CM pathogenesis. By measuring barrier integrity, endothelial activation, and transcriptional activity, we found that TNF-α and P. falciparum elicit distinct effects on HBMECs. Specifically, we observed P. falciparum induced a strong disruption of the endothelial barrier integrity with dampened endothelial activation. On the other hand, TNF-α was a potent activator of the endothelium, but only moderately disrupted HBMEC barrier integrity. Divergent transcriptomic profiles induced by TNF-α and P. falciparum in HBMECs substantiate the differential effects observed at the endothelial cellular level. These data suggest that P. falciparum is the critical factor driving the disruption of the BBB, while TNF-α is largely responsible for the endothelial inflammatory response. Targeted strategies that inhibit P. falciparum-induced signaling in endothelial cells could protect the integrity of the BBB and constitute the basis of novel therapies for CM.

## RESULTS

### Surface markers of endothelial activation increase in response to TNF-α, but not to P. falciparum.

**FIG 1:**
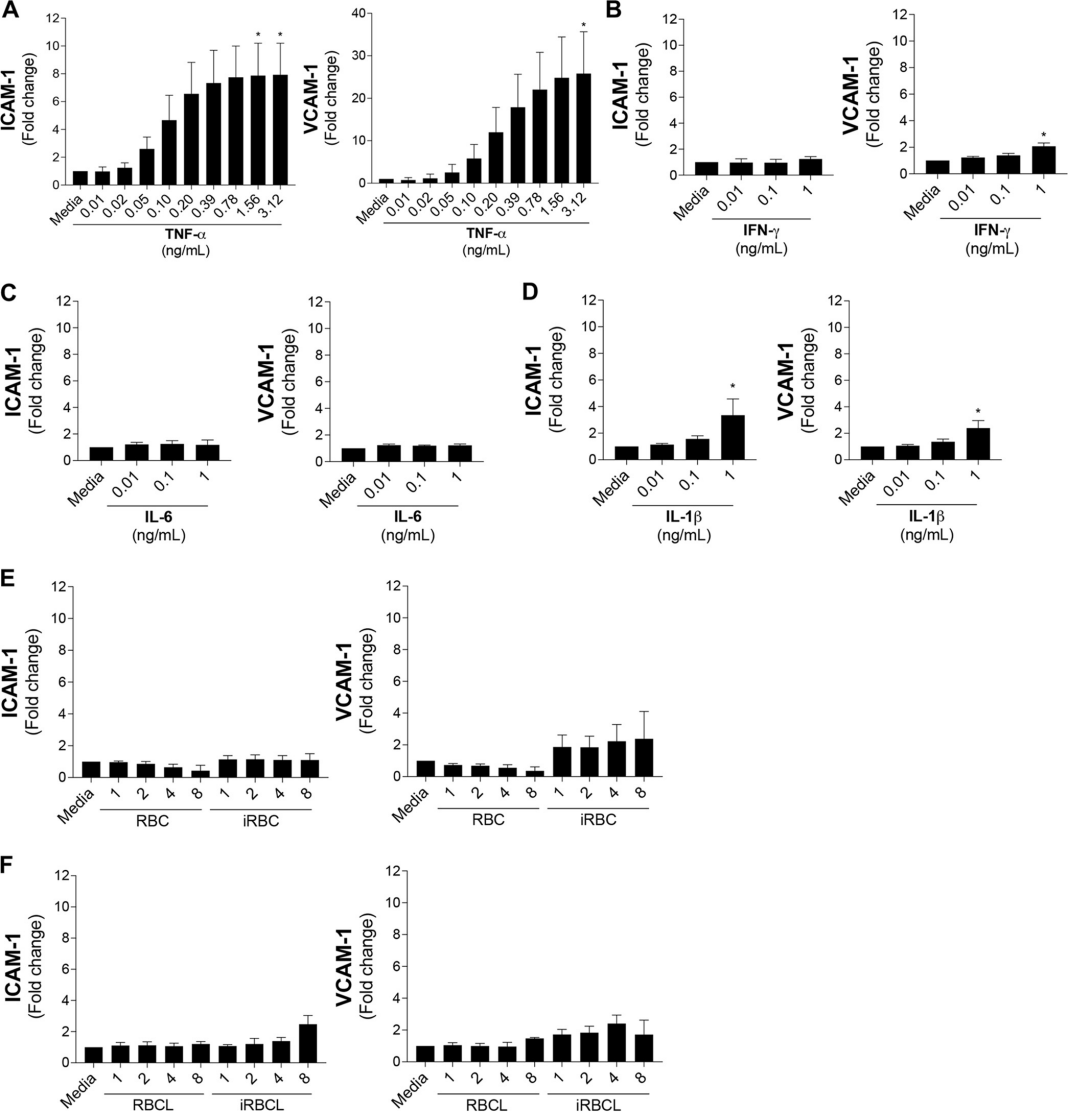
TNF-α, but not P. falciparum-iRBC lysates, significantly increases surface expression of ICAM-1 and VCAM-1 in HBMECs. HBMEC monolayers were incubated for 20 to 24 h with the indicated stimuli before determination of surface ICAM-1 or VCAM-1 levels (expressed as fold change over medium control). (A) TNF-α, (B) IFN-γ, (C) IL-6, and (D) IL-1β were tested at a range of physiological concentrations. (E and F) The dosages of P. falciparum-infected red blood cells (iRBCs), iRBC lysates (iRBCLs), uninfected red blood cells (RBCs), and RBC lysates (RBCLs) are expressed as number of RBCs or iRBCs (×10^6^) per surface area (square centimeters) of the cell culture well. Results are the average from 3 independent experiments with standard deviations. Statistical significance was determined by the Friedman test with Dunn’s multiple-comparison test (*, *P* < 0.05).

The activation of the endothelium is characterized by the increase of leukocyte adhesion molecules, like ICAM-1 and vascular cell adhesion protein-1 (VCAM-1) (26). Since both inflammatory cytokines and P. falciparum have been proposed to contribute to endothelial activation during malaria, changes in the surface expression of ICAM-1 and VCAM-1 were measured in a well-characterized human brain microvascular endothelial cell (HBMEC) line (7) after incubation with different cytokines and intact or lysed P. falciparum-infected red blood cells (iRBCs). As previously described (9, 10), TNF-α, a cytokine that is highly elevated in the circulation of cerebral malaria patients (0.001 to1 ng/mL), increases the expression of ICAM-1 and VCAM-1 in HBMEC monolayers in a dose-dependent manner (Fig. 1A). Similar results were observed in primary human brain microvascular endothelial cells (see Fig. S1A in the supplemental material). Other inflammatory cytokines, such as interferon gamma (IFN-γ), interleukin-6 (IL-6), and IL-1β, are also elevated in adults and children with cerebral malaria, with circulating concentrations ranging between 0.01 and 1 ng/mL (9, 10). In contrast to TNF-α, these cytokines do not strongly increase ICAM-1 or VCAM-1 surface expression (Fig. 1B to D).

**FIG 2:**
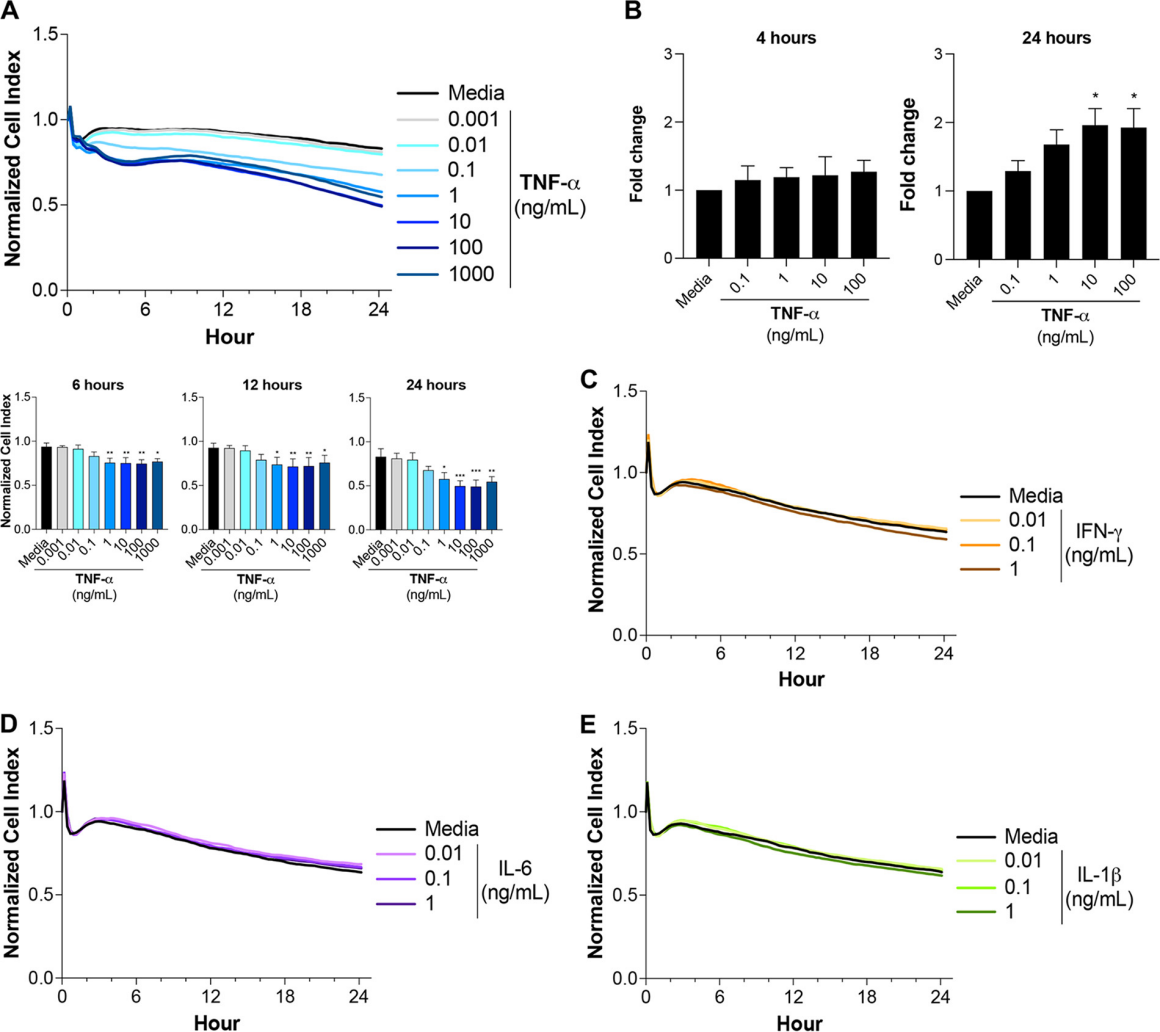
TNF-α moderately increases the permeability of HBMEC monolayers. The changes in HBMEC monolayer integrity were determined using (A and C to E) xCELLigence RTCA Biosensor technology or (B) leakage of FITC-dextran in a filter assay after addition of increasing concentrations of (A and B) TNF-α, (C) IFN-γ, (D) IL-6, or (E) IL-1β. Cell index values are proportional to the monolayer integrity. Results are expressed as fold change relative to the medium control and represent the average from at least 3 independent experiments with standard deviations. Statistical significance was determined by (A) the Kruskal-Wallis test or (B) the Friedman test with Dunn’s multiple-comparison test (*, *P* < 0.05; **, *P* < 0.01; ***, *P* < 0.001).

10.1128/mbio.01746-22.1 FIG S1 P. falciparum-iRBC lysates do not significantly increase surface ICAM-1, but strongly disrupt barrier integrity of primary human brain microvascular endothelial cells. Surface ICAM-1 and VCAM-1 levels were measured in primary human brain microvascular endothelial cells incubated with (A) TNF-α or (B) uninfected RBC lysates (RBCLs) and P. falciparum-iRBCLs (iRBCLs) for 24 h (8 × 10^6^ RBCLs or iRBCLs/cm^2^). Data are expressed as fold change over the medium control. The changes in HBMEC barrier integrity in response to TNF-α (C), RBCLs or iRBCLs (8 × 10^6^ RBCLs or iRBCLs/cm^2^) (D) were measured using xCELLigence RTCA Biosensor technology. Results are the average of 3 independent experiments with standard deviations. Statistical significance was determined by (B) the Friedman test or (C and D) the Kruskal-Wallis test with Dunn’s multiple-comparison test (*, *P* < 0.05; **, *P* < 0.01; ***, *P* < 0.001; ****, *P* < 0.0001). Figure S1 is related to Fig. 1 and 2. Download FIG S1, TIF file, 2.9 MB. Copyright © 2022 Zuniga et al. 2022 Zuniga et al. https://creativecommons.org/licenses/by/4.0/ This content is distributed under the terms of the Creative Commons Attribution 4.0 International license.

In contrast to TNF-α, incubation of HBMECs with P. falciparum-iRBCs at the schizont stage or with their lysates did not induce significant increases in markers of endothelial activation. The maximal concentration of iRBCs used (8 × 10^6^ iRBCs/cm^2^) represents the amount necessary to cover the surface of the well at double-layer density (see Materials and Methods), mimicking the situation *in vivo* where iRBCs are sequestered in brain capillaries (6). Despite the high concentrations of iRBCs that ruptured and released their contents over the HBMEC monolayer during the time of incubation of the assay, no significant increases in ICAM-1 or VCAM-1 surface expression were observed (Fig. 1E). No increases were also observed after incubation with lysates of iRBCs at the same concentrations in HBMECs (Fig. 1F) or in primary human brain microvascular endothelial cells (Fig. S1B). Taken together, these results indicate strong differences in the effects of TNF-α and P. falciparum on endothelial activation.

### HBMEC barrier integrity is strongly disrupted by P. falciparum.

P. falciparum and inflammatory cytokines have both been implicated in the disruption of endothelial barrier integrity (27, 28), but their relative contributions are not clearly understood. To better understand the effects of inflammatory cytokines and P. falciparum on HBMEC barrier integrity over time, we measured the monolayer’s impedance utilizing xCELLigence technology. TNF-α reduced barrier integrity in a dose-dependent manner, with physiological concentrations such as 1 ng/mL (10) causing small, but significant decreases over 24 h (Fig. 2A). In parallel, a filter permeability assay showed similar effects of TNF-α on endothelial integrity (Fig. 2B). Similar results were observed in primary human brain microvascular endothelial cells (Fig. S1C). Physiological concentrations of IFN-γ, IL-6, and IL-1β did not affect HBMEC barrier integrity over 24 h (Fig. 2C to E). In contrast, conditioned medium from lipopolysaccharide (LPS)-stimulated human peripheral blood mononuclear cells (PBMCs), which contains a mixtue of cytokines, including high levels of TNF-α, similarly affected HBMEC barrier integrity (Fig. S2).

10.1128/mbio.01746-22.2 FIG S2 Conditioned medium from LPS-stimulated PBMCs affects HBMEC barrier integrity. Human peripheral blood mononuclear cells (PBMCs) were incubated with medium or LPS (1 μg/mL) for 24 h. (A) Levels of inflammatory cytokines in supernatants of PBMC cultures; (B) effects of PBMC-stimulated supernatants on HBMEC barrier integrity using xCELLigence. LPS alone (1 μg/mL) (LPS No Cells) was used as a control. Results are the average from 3 independent experiments with standard deviations. Statistical significance was determined by (A) unpaired *t* test or (B) the Kruskal-Wallis test with Dunn’s multiple-comparison test (*, *P* < 0.05; **, *P* < 0.01; ***, *P* < 0.001). Figure S2 is related to Fig. 1. Download FIG S2, TIF file, 2.9 MB. Copyright © 2022 Zuniga et al. 2022 Zuniga et al. https://creativecommons.org/licenses/by/4.0/ This content is distributed under the terms of the Creative Commons Attribution 4.0 International license.

**FIG 3:**
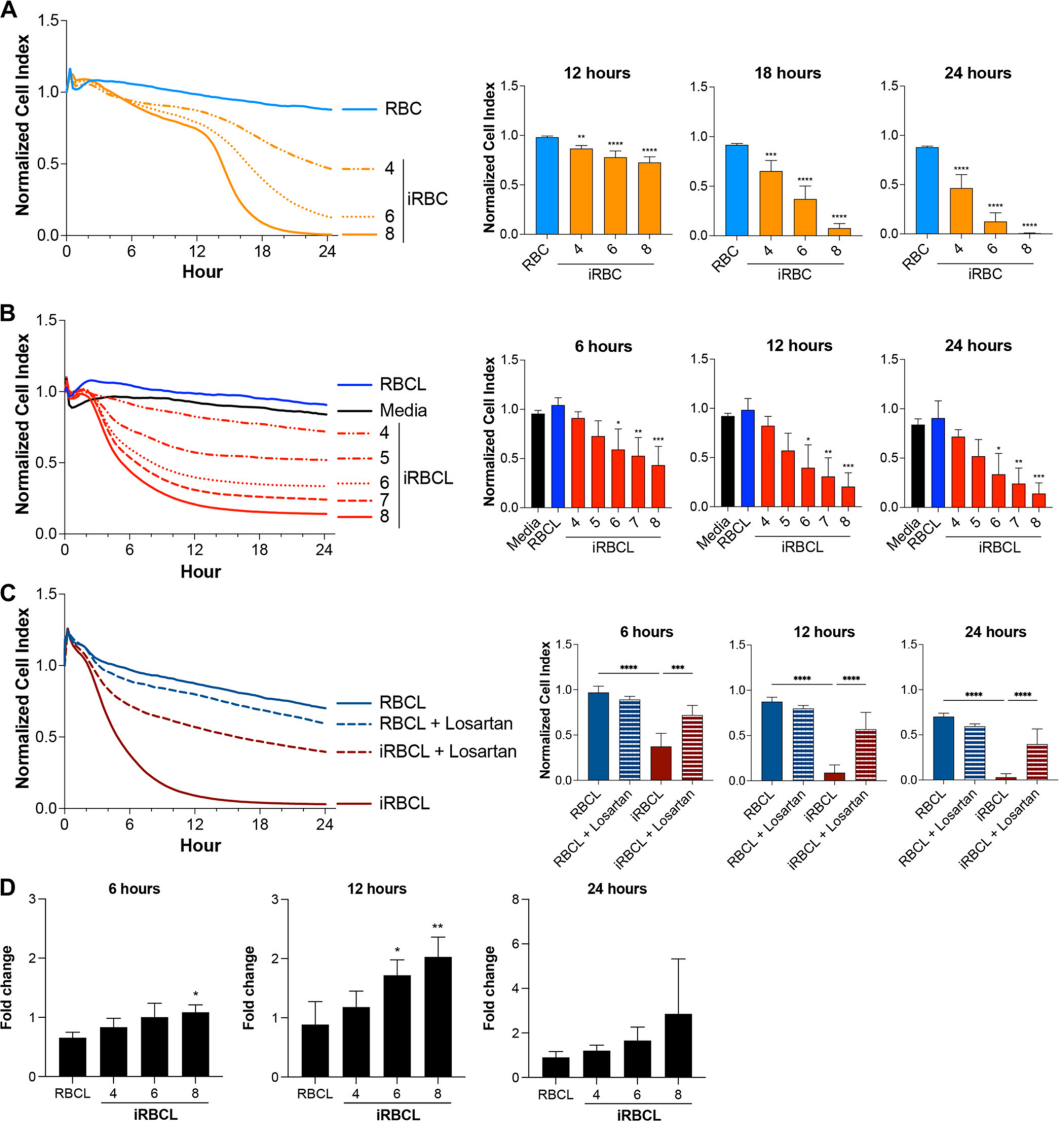
P. falciparum-iRBCs and their lysates strongly increase the permeability of HBMEC monolayers. The changes in HBMEC monolayer integrity in response to different dosages of (A) intact iRBCs in the schizont stage or (B to D) their lysates (iRBCLs) were measured using (A to C) xCELLigence RTCA Biosensor technology or (D) the leakage of FITC-dextran in a filter assay. The concentrations are expressed as the number of RBCs or iRBCs (×10^6^) per surface area (square centimeters) of the cell culture well. (A) Uninfected RBCs (8 × 10^6^/cm^2^) or (B to D) their lysates (RBCLs) (8 × 10^6^/cm^2^) from the same blood donation were included as a control. (C) HBMEC monolayers were preincubated with 200 μM losartan for 1 h prior to addition of the lysates (8 × 10^6^/cm^2^). (D) The leakage of FITC-dextran is expressed as fold change relative to the medium control. Results represent the average from 3 independent experiments with standard deviations. Statistical significance was determined by (A, C, and D) one-way ANOVA with Tukey’s multiple-comparison test or (B) the Kruskal-Wallis test with Dunn’s multiple-comparison test (*, *P* < 0.05; **, *P* < 0.01; ***, *P* < 0.001; ****, *P* < 0.0001).

To study the effect of P. falciparum on HBMEC barrier integrity, the effects of different concentrations of intact iRBCs and lysed iRBCs (iRBCLs) were compared, using uninfected RBCs and their lysates as controls. Both iRBCLs and intact iRBCs strongly disrupt HBMEC barrier integrity in a dose-dependent manner, with significant decreases in HBMEC barrier integrity detected already at 4 × 10^6^/cm^2^, a concentration that results in a monolayer of iRBCs over the surface of the well (Fig. 3A and B). A marked delay in the effect of intact iRBCs compared to iRBCLs was observed, probably reflecting the time needed for iRBCs to spontaneously rupture and release their contents, a process necessary for the iRBC-induced HBMEC barrier disruption (7). A similar iRBCL dose-dependent loss of barrier integrity was observed when primary human brain microvascular endothelial cells were used in the assay (Fig. S1D).

The effect of iRBCLs was observed after 3 to 4 h of incubation, which suggests that signaling induced by iRBCLs in HBMECs could mediate the loss of barrier integrity. We also confirmed that iRBCL-induced HBMEC barrier disruption can be significantly inhibited by pretreatment with losartan (an inhibitor of angiotensin receptor 1), as described previously for intact iRBCs (7), further suggesting that specific signaling triggered by iRBCLs, and not unspecific toxicity, could induce HBMEC barrier disruption (Fig. 3C). A parallel filter permeability assay confirmed similar effects of iRBCLs on HBMEC barrier function (Fig. 3D). Taken together, these results show different intensities and kinetics of endothelial barrier disruption induced by TNF-α and P. falciparum. These results also support that the multiwell xCELLigence permeability assay may be used as a first-line screening tool to identify candidate endothelial protective drugs.

### P. falciparum induces low levels of cell death in HBMECs.

**FIG 4:**
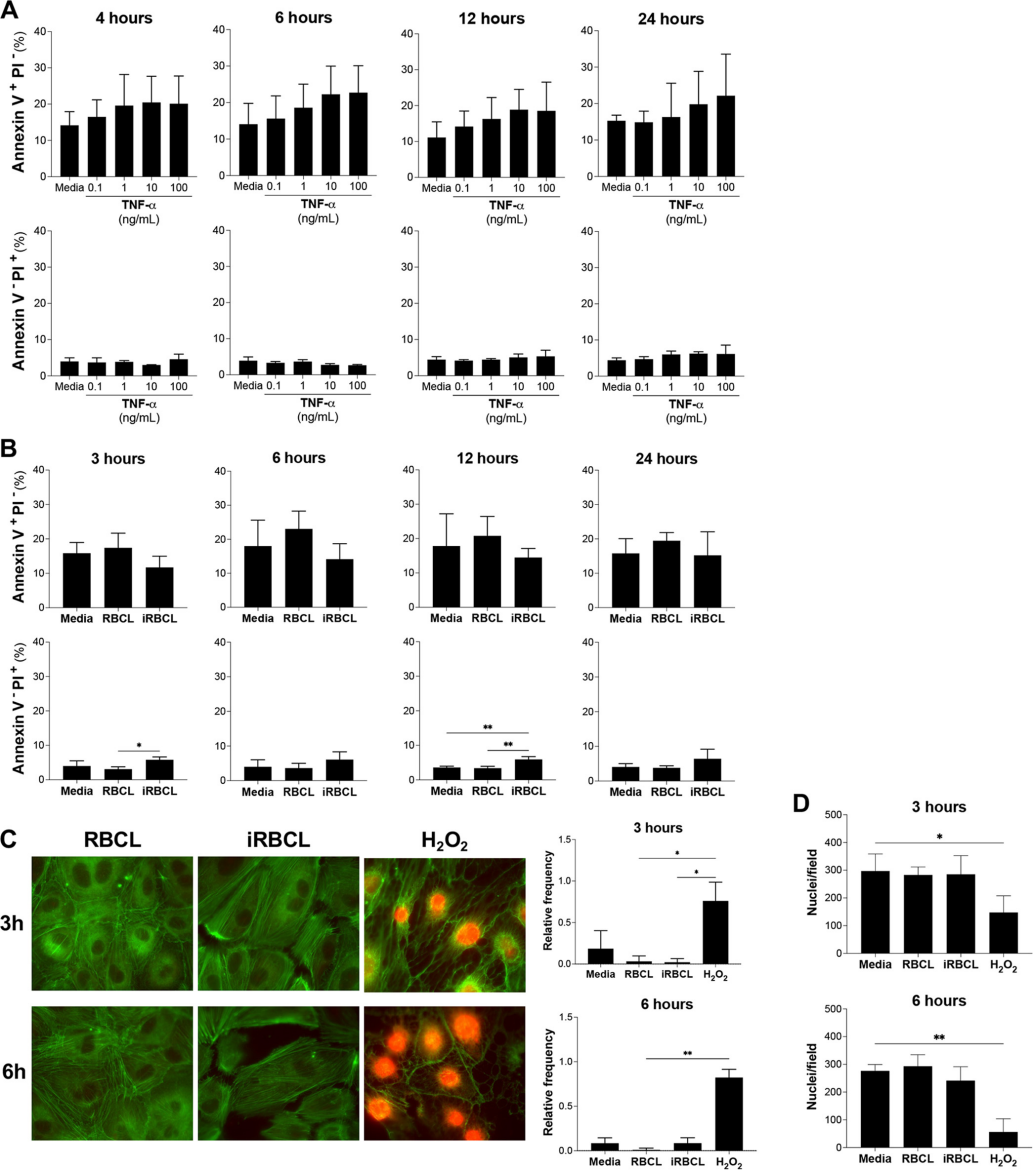
P. falciparum-iRBC lysates do not induce apoptosis in HBMECs. HBMEC monolayers were incubated with increasing concentrations of (A) TNF-α or (B) RBCLs and iRBCLs (8 × 10^6^/cm^2^) for the indicated times. (A and B) The presence of early apoptotic (annexin V^+^ PI^−^) and necrotic (annexin V^−^ PI^+^) populations was detected by flow cytometry. (C and D) HBMEC monolayers were incubated with RBCLs (8 × 10^6^/cm^2^), iRBCLs (8 × 10^6^/cm^2^), or H_2_O_2_ (1 mM) as a positive control and stained for F-actin (green) and PI (red) to detect necrotic cells (C) or PI and nuclear staining to quantify the number of HBMECs that remain attached to the coverslip (D) at 3 and 6 h after treatment. (C) Representative immunofluorescent images are shown. The relative frequency of PI^+^ cells (C) and the average number of nuclei per field from 3 fields (D) from 2 independent experiments are shown and include the medium control. (A and B) Results are the average from 3 independent experiments with standard deviations. Statistical significance was determined by one-way ANOVA with Tukey’s multiple-comparison test (B) or the Kruskal-Wallis test with Dunn’s multiple-comparison test (C and D) (*, *P* < 0.05; **, *P* < 0.01).

To determine whether TNF-α and P. falciparum-induced HBMEC barrier disruption is mediated by the onset of apoptosis, the quantification of early and late apoptotic and necrotic cell populations was first set up in HBMECs (Fig. S3). HBMEC monolayers incubated with TNF-α presented an increase in early apoptotic cells (annexin V^+^ propidium iodide [PI]^−^) starting at 4 h, although it was not statistically significant (Fig. 4A). Similarly, late apoptotic HBMEC populations increase at 12 h, as cellular membranes become compromised (annexin V^+^ PI^+^) (Fig. S4A). The appearance of these populations suggests that TNF-α induces apoptosis in a fraction of cells (Fig. 2A). Similar results have been described before with TNF-α inducing apoptosis in other types of endothelial cells (16, 29).

10.1128/mbio.01746-22.3 FIG S3 Camptothecin induces apoptosis in HBMEC monolayers. (A to D) HBMECs were incubated with camptothecin (10 μM) or hydrogen peroxide (H_2_O_2_) (1 mM) for 4 h. (A) Live (annexin V^−^ PI^−^), (B) necrotic (annexin V^+^ PI^+^), (C) early apoptotic (annexinV^+^ PI^−^), and (D) late apoptotic (annexin V^+^ PI^+^) populations were detected with APC-conjugated annexin V and propidium iodide (PI). Results are the average from 3 independent experiments with standard deviations. Statistical significance was determined by one-way ANOVA with Tukey’s multiple-comparison test (**, *P* < 0.01; ***, *P* < 0.001; ****, *P* < 0.0001). Figure S3 is related to Fig. 4. Download FIG S3, TIF file, 2.9 MB. Copyright © 2022 Zuniga et al. 2022 Zuniga et al. https://creativecommons.org/licenses/by/4.0/ This content is distributed under the terms of the Creative Commons Attribution 4.0 International license.

10.1128/mbio.01746-22.4 FIG S4 P. falciparum-iRBCLs do not significantly induce the increase of late apoptotic populations. (A) HBMEC monolayers were incubated with increasing concentrations of TNF-α, (B) uninfected RBC lysates (RBCLs) (8 × 10^6^/cm^2^) or P. falciparum-iRBCLs (8 × 10^6^/cm^2^) for the indicated times. Viable (annexin V^−^ PI^−^) and late apoptotic (annexin V^+^ PI^+^) populations were detected with APC-conjugated annexin V and propidium iodide (PI). Results represent the average of 3 independent experiments. Figure S4 is related to Fig. 4. Download FIG S4, TIF file, 2.9 MB. Copyright © 2022 Zuniga et al. 2022 Zuniga et al. https://creativecommons.org/licenses/by/4.0/ This content is distributed under the terms of the Creative Commons Attribution 4.0 International license.

To determine whether P. falciparum-iRBCLs induce apoptosis, HBMEC monolayers were incubated with the iRBCL concentration that induced the strongest barrier disruption (8 × 10^6^ iRBCs/cm^2^) (Fig. 3B). Unlike TNF-α, iRBCLs did not induce an increase in early apoptotic cells at any time (Fig. 4B), indicating that the observed loss of barrier integrity induced by iRBCLs (Fig. 3B) is not caused by a generalized apoptotic response. However, there was a small increase in necrotic cell populations (annexinV^−^ PI^+^) at all time points (Fig. 4B). To further analyze these necrotic cells, nuclear staining of PI was detected through immunofluorescence microscopy. No nuclear PI staining could be observed in HBMECs at 3 or 6 h after incubation with iRBCLs (Fig. 4C), suggesting that the slight increase in necrotic cell populations (annexin V^−^ PI^+^) observed through flow cytometry could be in part a consequence of the trypsinization step necessary to collect these cells and would imply that incubation with iRBCLs left HBMECs more susceptible to necrosis. To estimate the relative contribution of the small levels of HBMEC cell death to the disruption of endothelial barrier integrity, we used TNF-α as a reference, since it is well known that TNF-α is a potent inducer of apoptosis at high concentrations (100 ng/mL) (16). We observed that after 3 h of incubation with iRBCLs, there is no detectable increase in HBMEC cell death (Table S1), but there is already a significant loss of barrier integrity, as evidenced by a 19% decrease in the cell index (Fig. 3). After 6 h of incubation, TNF-α induced 3.8 times more cell death, but 2.2 times less barrier disruption than iRBCLs, which corresponds to iRBCLs being 8.3 times more disruptive than what would be predicted based in the observed increase in cell death at this time (Table S1). These estimates suggest that although HBMEC cell death may contribute to the loss of endothelial barrier integrity by iRBCLs, it does not appear to be the major cause driving it.

10.1128/mbio.01746-22.8 TABLE S1 Comparison of cell death versus loss of barrier integrity by TNF-α and iRBCLs in HBMECs. Download Table S1, DOCX file, 0.01 MB. Copyright © 2022 Zuniga et al. 2022 Zuniga et al. https://creativecommons.org/licenses/by/4.0/ This content is distributed under the terms of the Creative Commons Attribution 4.0 International license.

Actin cytoskeleton patterns typical of cells detaching from each other were observed in HBMECs incubated with iRBCLs at 3 and 6 h (Fig. 4C), when loss of endothelial monolayer integrity is detected (Fig. 3B), but no significant cell detachment is observed at these time points (Fig. 4D). Similar results were observed in primary human brain microvascular endothelial cells (Fig. S5). Taken together, these results indicate that although iRBCLs induce a small percentage of cell death, the loss of HBMEC barrier integrity is probably driven by the activation of specific signaling pathways that destabilized interendothelial cell junctions in the monolayer.

10.1128/mbio.01746-22.5 FIG S5 P. falciparum-iRBCLs do not induce apoptosis in primary human brain microvascular endothelial cells. Monolayers of primary human brain microvascular endothelial cells were incubated with (A to D) TNF-α or (E to I) uninfected RBCLs (8 × 10^6^/cm^2^) and P. falciparum-iRBCLs (8 × 10^6^/cm^2^) for the indicated times. The presence of (A and E) early apoptotic (annexin V^+^ PI^−^), (B and F) necrotic (annexin V^−^ PI^+^), (C and G) late apoptotic (annexin V^+^ PI^+^), and (D and H) viable populations was detected through flow cytometry. (I) The presence of necrotic cells was confirmed through immunofluorescence by PI and nuclear stain. Data are expressed as the percentage of nuclei positive for PI staining. (A to H) Results are the average from 3 independent experiments with standard deviations. Statistical significance was determined by (F) one-way ANOVA with Tukey’s multiple-comparison test or (G) Kruskal-Wallis test with Dunn’s multiple-comparison test (*, *P* < 0.05). Figure S5 is related to Fig. 4. Download FIG S5, TIF file, 2.9 MB. Copyright © 2022 Zuniga et al. 2022 Zuniga et al. https://creativecommons.org/licenses/by/4.0/ This content is distributed under the terms of the Creative Commons Attribution 4.0 International license.

### HBMECs display distinct transcriptional profiles upon exposure to TNF-α and P. falciparum-iRBCLs.

**FIG 5:**
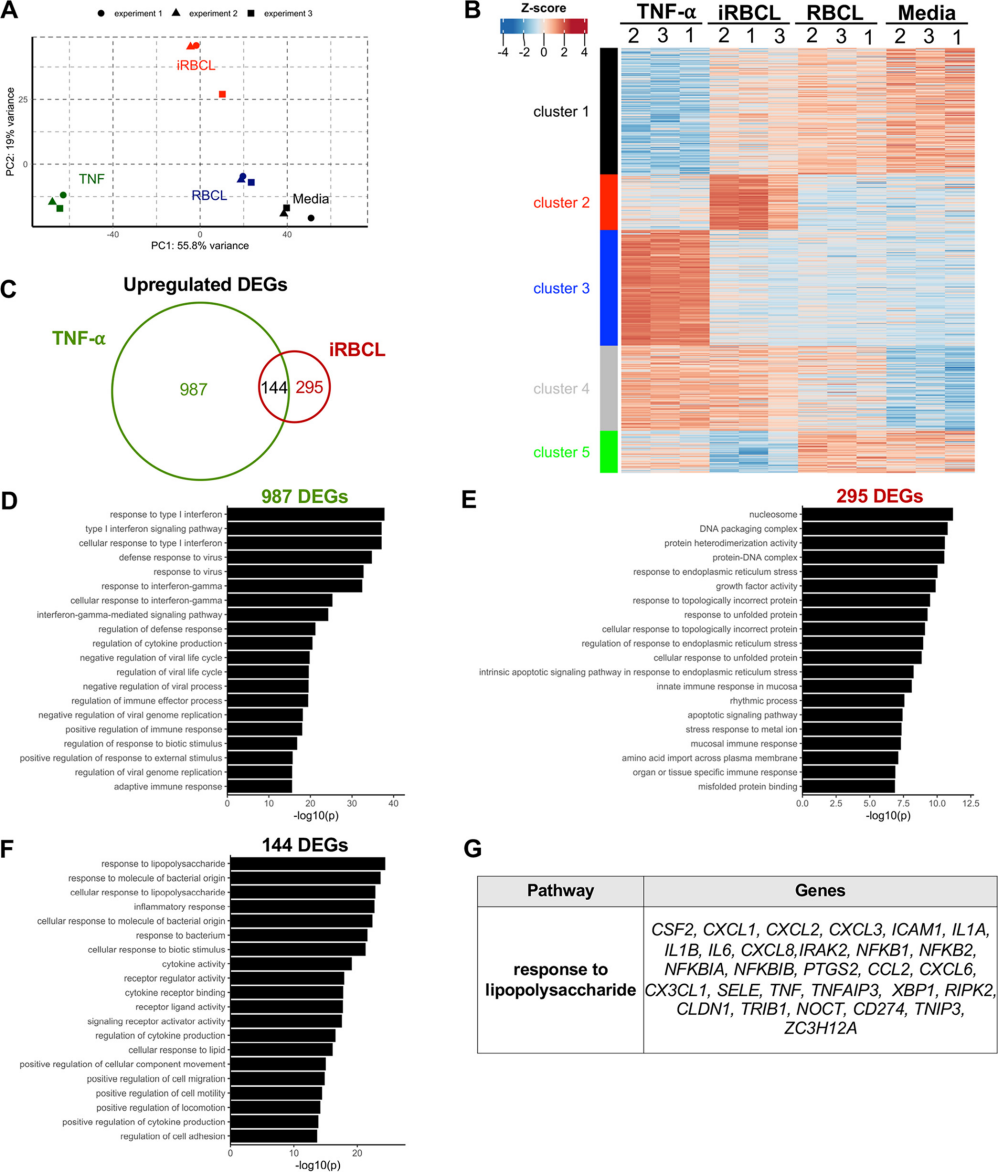
TNF-α and P. falciparum-iRBC lysates induce distinct transcriptional profiles, with overlap in genes associated with inflammation. HBMEC monolayers were incubated with medium, TNF-α (1 ng/mL), RBCLs (8 × 10^6^/cm^2^), or P. falciparum-iRBCLs (8 × 10^6^/cm^2^) for 6 h prior to RNA extraction and sequencing. (A) Principal-component analysis of 2,373 differentially expressed genes (DEGs). Sample points correspond to independent experiments. (B) Heat map of the 2,373 DEGs with *K-*means clustering to distinguish uniquely regulated sets of genes. Experimental samples for all conditions are shown and denoted with 1, 2, or 3. (C) Overlap analysis of TNF-α versus medium (TNF-α) and iRBCL versus RBCL (iRBCL) DEGs. Cutoffs of DEGs were set to log_2_ fold change of >1 or <−1 with an adjusted *P* value of <0.05. (D to F) Gene Ontology (GO) term enrichment analysis of DEGs from panel C. (D to F) Top 20 pathways mapped by upregulated DEGs (D) unique to TNF-α, (E) unique to iRBCLs, and (F) shared by both. (G) Gene list of the top pathway from panel F.

To further characterize the effects of P. falciparum-iRBCLs and TNF-α on HBMEC monolayers, transcriptional changes were determined by RNA sequencing in 3 independent experiments. Principal-component analysis using differentially expressed genes (DEGs) (*n* = 2,373) demonstrates the reproducibility of our experiments and indicates that P. falciparum-iRBCLs and TNF-α induce very different transcriptional profiles in HBMECs (Fig. 5A). Conversely, samples of HBMECs exposed to medium or uninfected red blood cell lysates (RBCLs) clustered closely, suggesting they have similar transcriptional profiles. The transcriptional differences upon TNF-α or P. falciparum-iRBCL exposure were further investigated by hierarchical clustering analysis of DEGs, which groups them into 5 uniquely regulated clusters visualized by a heat map (Fig. 5B). Out of the 5 clusters, cluster 4 is the only one to contain genes regulated similarly by TNF-α and P. falciparum-iRBCLs, which highlights the differences in transcriptional responses to these two factors.

We next performed supervised comparisons to identify genes that are significantly up- and downregulated relative to their respective controls. We then determined the unique and overlapping upregulated genes in response to TNF-α and P. falciparum-iRBCLs. Overall, TNF-α uniquely upregulated (*n* = 987) and downregulated (*n* = 707) more genes than P. falciparum-iRBCLs (Fig. 5C; Fig. S6A). Pathway enrichment analysis of genes uniquely upregulated by TNF-α indicates roles in the regulation of inflammatory responses (Fig. 5D). Many of these genes encode proteins that mitigate the signal transduction regulating these inflammatory responses. For example, *TRAF2* and *TRAF3* encode TNF receptor-associated factors (TRAFs), which are responsible for transducing downstream signaling of immune receptors and regulating the inflammatory responses associated with NF-κB (Table S1) (30). There is also upregulation of interferon regulatory factors (IRFs), which are thought to regulate the signaling of pattern recognition receptors (Table S2A) (31). In contrast, genes uniquely upregulated (*n* = 295) by P. falciparum-iRBCLs are enriched in pathways that broadly fall under the endoplasmic reticulum (ER) stress response (Fig. 5E; Table S2B). However, P. falciparum-iRBCLs also induced an increase of inflammatory response-related genes that are shared with TNF-α and include *ICAM1*, a marker of endothelial activation (Fig. 5F and G; Table S2C). Overall, these data demonstrate that P. falciparum-iRBCLs and TNF-α have fundamentally different effects on HBMEC monolayers at the transcriptional level, inducing preferentially ER stress and inflammatory responses, respectively.

10.1128/mbio.01746-22.6 FIG S6 DEGs uniquely downregulated by P. falciparum-iRBCLs map to pathways associated with lipid processes. (A) Overlap analysis of TNF-α versus medium (TNF-α) and iRBCL versus RBCL (iRBCL) DEGs. Cutoffs of DEGs were set to log_2_ fold change of >1 or <−1 with an adjusted *P* value of <0.05. (B) Gene Ontology (GO) term analysis of DEGs uniquely downregulated by iRBCLs. The top 20 pathways are shown. No significant pathways were observed with downregulated DEGs unique to TNF-α or those shared by TNF-α and iRBCLs. (C) Gene lists of the top 20 pathways from (B). Figure S6 is related to Fig. 5. Download FIG S6, TIF file, 2.9 MB. Copyright © 2022 Zuniga et al. 2022 Zuniga et al. https://creativecommons.org/licenses/by/4.0/ This content is distributed under the terms of the Creative Commons Attribution 4.0 International license.

10.1128/mbio.01746-22.9 Table S2 (A) Gene lists of top 20 pathways mapped by DEGs uniquely upregulated by TNF-α in HBMECs. (B) Gene lists of top 20 pathways mapped by DEGs uniquely upregulated by P. falciparum-iRBCLs in HBMECs. (C) Gene lists of top 20 pathways mapped by DEGs upregulated by TNF-α and P. falciparum-iRBCLs in HBMECs. Download Table S2, DOCX file, 0.02 MB. Copyright © 2022 Zuniga et al. 2022 Zuniga et al. https://creativecommons.org/licenses/by/4.0/ This content is distributed under the terms of the Creative Commons Attribution 4.0 International license.

Pathway enrichment analysis focused on interendothelial junction structural components and regulators of junction integrity was performed to identify signaling pathways modulated by iRBCLs compared to RBCLs (Table S3). This analysis identified specific upregulated pathways, such as vascular endothelial growth factor (VEGF) and endothelial nitric oxide synthase (eNOS), but also downregulated pathways, such as RhoA and focal adhesion kinase (FAK), which are all known to regulate BBB integrity (32–34), suggesting possible mechanisms for the iRBCL-induced loss of barrier integrity of endothelial cells.

10.1128/mbio.01746-22.10 TABLE S3 List of interendothelial junction-related pathways regulated by P. falciparum-iRBCLs in HBMECs. Download Table S3, DOCX file, 0.02 MB. Copyright © 2022 Zuniga et al. 2022 Zuniga et al. https://creativecommons.org/licenses/by/4.0/ This content is distributed under the terms of the Creative Commons Attribution 4.0 International license.

To obtain more insight into the distinct features of P. falciparum-iRBCL effects on cellular activation, we decided to compare our transcriptome sequencing (RNA-seq) transcriptional profiling results with previously published data on the effects of P. falciparum-iRBCs on an immune cell type, myeloid dendritic cells (mDCs) (35). We observed that the number of regulated genes induced by iRBCs in mDCs is significantly larger than that in HBMECs, probably reflecting the large quantity of immune-related pathways activated by iRBCs in mDCs (35) that are not present in endothelial cells. We also observed that the majority of modulated genes induced by iRBCs in HBMECs are unique to this cell type and are not found in mDCs (Fig. S7).

10.1128/mbio.01746-22.7 FIG S7 DEGs by P. falciparum-iRBCs are largely different in mDCs and HBMECs. Shown are results from overlap analysis of uniquely regulated DEGs from myeloid dendritic cells (mDCs) and HBMECs stimulated with P. falciparum-iRBCs and -iRBCLs, respectively. Cutoffs of DEGs were log_2_ fold change of >1 or <−1 with an adjusted *P* value of <0.05. Figure S7 is related to Fig. 5. Download FIG S7, TIF file, 2.9 MB. Copyright © 2022 Zuniga et al. 2022 Zuniga et al. https://creativecommons.org/licenses/by/4.0/ This content is distributed under the terms of the Creative Commons Attribution 4.0 International license.

### Discrepancies in mRNA versus protein expression levels in different cytokines and chemokines upon HBMEC exposure to P. falciparum.

**FIG 6:**
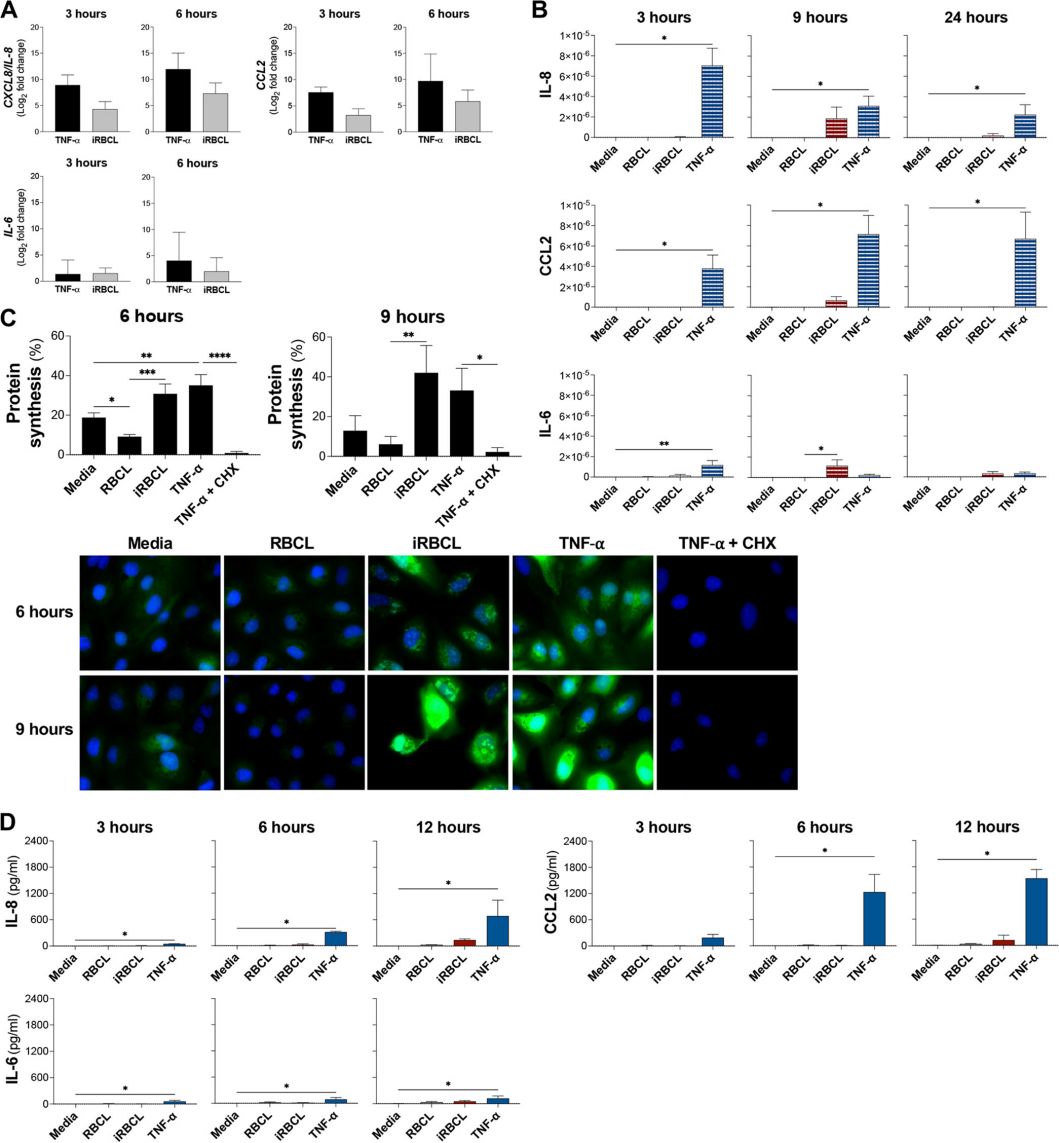
TNF-α and P. falciparum-iRBC lysates induce transcriptionally similar, but translationally different levels of cytokines and chemokines. (A to D) HBMEC monolayers were incubated with media, TNF-α (1 ng/mL), RBCLs (8 × 10^6^/cm^2^), or P. falciparum-iRBCLs (8 × 10^6^/cm^2^) for the indicated times. (A) Quantitative PCR for each time point was conducted to determine the expression levels of *IL8*, *CCL2*, and *IL6*. The fold change of TNF-α relative to medium (black bars) and iRBCLs relative to RBCL (gray bars) is shown. (B) Lysates of HBMECs after the incubation were used to determine intracellular protein levels of IL-8, CCL2, and IL-6. Cytokine and chemokine levels were normalized to the total protein level of the cell lysate. (C) Changes in protein synthesis were determined by the incorporation of Click-iT HPG-Alexa Fluor 488 measured by flow cytometry and visualized by immunofluorescence microscopy. Representative images are shown. HPG is in green, and nuclei are in blue. TNF-α with cycloheximide (CHX) (100 μg/mL) was included as a control. (D) Medium of HBMEC cultures was collected to determine secreted protein levels of IL-8, CCL2, and IL-6. Results are the average from at least 3 independent experiments with standard deviation. Statistical significance was determined by (C) one-way ANOVA with Tukey’s multiple-comparison test or (A, B, and D) Kruskal-Wallis test with Dunn’s multiple-comparison test (*, *P* < 0.05; **, *P* < 0.01; ***, *P* < 0.001; ****, *P* < 0.0001).

TNF-α activation of the endothelium induces the secretion of inflammatory cytokines and chemokines, along with the increase in surface leukocyte adhesion molecules (13, 36). The RNA-seq analysis revealed that upregulation of specific inflammatory response pathways is induced by both TNF-α and P. falciparum-iRBCLs (Fig. 5F). As a means to validate the RNA-seq analysis and to better understand the state of HBMEC activation in response to TNF-α and P. falciparum-iRBCLs, we first confirmed the upregulation of IL-8, CCL2, and IL-6 mRNA identified by the RNA-seq analysis (Fig. 5G). Quantitative PCR confirmed that both P. falciparum-iRBCLs and TNF-α transcriptionally induce comparable levels of *IL8*, *CCL2*, and *IL6* mRNA (Fig. 6A).

To determine whether the observed transcriptional profiles would be similar at the protein level, we first measured the total levels of IL-8, CCL2, and IL-6 in HBMECs. We observed that, despite inducing similar levels of transcription, TNF-α induced a greater increase of intracellular expression of IL-8 and CCL2 proteins than P. falciparum-iRBCLs. When stimulated by TNF-α, IL-8 appears to have an early induction that decays over time, while CCL2 levels remained high for 24 h. On the other hand, iRBCLs induced only limited amounts of IL-8 and CCL2 at 9 h, with virtually undetectable levels at other time points. IL-6 levels were low for both stimuli (Fig. 6B). To determine whether the reduced intracellular levels of IL-8, CCL2, and IL-6 induced by P. falciparum-iRBCLs may be the result of generalized impaired translation in HBMECs, we measured changes in total protein synthesis. We observed that P. falciparum-iRBCLs and TNF-α robustly induce similar levels of total protein synthesis in HBMECs (Fig. 6C), indicating that P. falciparum-iRBCLs do not induce a generalized impairment in translation and suggesting that the reduced protein levels of IL-8, CCL2, and IL-6 may be a result of post-transcriptional regulation, which has been described for specific cytokine genes (37).

Since regulation of cytokine secretion is another important pathway that determines the final levels of cytokines that are released into the circulation by endothelial cells (38), we compared the levels of secreted cytokines by HBMECs in response to TNF-α and iRBCLs. We observed that TNF-α induces significantly greater secretion of IL-8 and CCL2 compared to P. falciparum-iRBCLs, especially at late time points, as would be expected by the observed higher intracellular levels of these chemokines. Accordingly, IL-6 was weakly secreted after incubation with TNF-α or P. falciparum-iRBCLs (Fig. 6D), matching the low intracellular levels of this cytokine.

Overall, the differences indicate that TNF-α robustly triggers a classical endothelial activation profile with high levels of secreted cytokines and chemokines. On the other hand, P. falciparum does not induce strong expression at the protein level of these mediators, despite inducing comparable mRNA levels, ultimately resulting in low levels of secreted cytokines and chemokines.

## DISCUSSION

In this study, we found that TNF-α and P. falciparum-iRBCs have distinct effects on HBMECs, with a clear difference observed in their abilities to induce endothelial activation. Whereas TNF-α induced strong upregulation of surface ICAM-1 and VCAM-1 in HBMECs, as well as cytokine production, P. falciparum-iRBCs did not. Nonetheless, iRBCs strongly increase HBMEC permeability via a cell-death-independent mechanism, associated with a transcriptional response indicative of increased cellular stress. Our findings suggest that P. falciparum-iRBCs are a major contributor to endothelial barrier disruption, while TNF-α would largely contribute to CM by inducing endothelial activation, upregulating the expression of ICAM-1 that can promote the sequestration of iRBCs. The resulting accumulation of iRBCs in the confined space of the brain capillaries blocks perfusion (39), which would allow for high concentrations of parasite-derived factors released upon iRBC rupture to cause increased endothelial permeability.

The loss of BBB integrity is a crucial step in the pathogenesis of CM, ultimately leading to brain swelling and death. However, very little is known about the mechanisms underlying this disruption. Cytoadhesion of iRBCs to brain capillaries is thought to be a critical step leading to the disruption of the BBB, since specific PfEMP1 variants expressed on the surface of iRBCs that bind receptors on the brain endothelium, such as ICAM-1 and EPCR, are found in CM patients (5). In addition, dense sequestration of iRBCs is found near hemorrhage sites in the brains of deceased CM patients (6). Endothelial activation and loss of BBB integrity are observed during CM (40, 41), but the relative contribution of inflammatory cytokines, such as TNF-α, and/or sequestered iRBCs to these processes remains unclear. TNF activation is characterized by the secretion of inflammatory mediators and the upregulation of surface leukocyte adhesion molecules, such as ICAM-1 and VCAM-1, and can be followed by the onset of apoptosis upon sustained stimulation (26). TNF-α induced strong upregulation of surface ICAM-1 and VCAM-1 in HBMECs, as previously reported (13). However, our results also demonstrate that neither iRBCs nor their lysates induce this effect, which suggest parasite-derived factors are not activating HBMECs. Along the same lines, cytokine secretion was strongly induced by TNF-α, but not by iRBCLs. These effects reveal TNF-α induces a profile characteristic of endothelial activation, while P. falciparum-iRBCs and their lysates (iRBCLs) do not.

It was previously shown that the rupture of P. falciparum-iRBCs and release of their contents induced significant disruption in HBMEC barrier function, which was not observed with genetically engineered parasites unable to induce the rupture of the infected erythrocytes (7). In this study, we directly compared the effects of intact iRBCs and their lysates, finding similar strong effects in barrier disruption, while TNF-α only had a moderate effect on barrier integrity, further supporting a critical role for iRBC rupture and release of their contents in the disruption of endothelial barrier function. The concentrations of iRBCs and lysates used in our studies were matched to represent the brain capillaries densely packed with P. falciparum-iRBCs that are observed in patients that died of CM (42). At the highest concentration, which represents two layers of iRBCs over the HBMECs, the disruptive effect of iRBCLs on HBMEC barrier function was very strong, but significant disruption was also observed at lower concentrations representing one layer of iRBCs, which is found in smaller capillaries (6). Different strains of P. falciparum, such as 3D3 (used here), IT4var19, HB3var03, and ItGICAM1 (43), all with different cytoadherence characteristics, were able to efficiently induce disruption of barrier integrity in HBMECs *in vitro*. These results further suggest that when iRBCs are densely packed, as in brain capillaries where iRBCs cytoadhere, or under our *in vitro* conditions, the release of iRBC contents is critical in the disruption of endothelial barrier integrity. Under similar conditions, staining of interendothelial junction proteins in tight junctions, focal adhesions, and adherens junctions showed an extensive disruption of these structures, with cells detaching from each other (7).

Although we did not investigate the effects of iRBC binding to HBMECs through host EPCR or ICAM-1, a comparison of adherent P. falciparum-iRBCs in the trophozoite stage (binding to endothelial cells) versus the schizont stage (binding, but presumably also rupturing and releasing parasite-derived contents) found much greater barrier disruptive activity in the schizonts (43). Also, the incubation of schizonts from an EPCR binding strain induced similar endothelial barrier disruption compared to nonadherent schizonts (7). Taken together with our current observations, these suggest the rupture and release of contents occurring in the mature schizont stage of iRBC are a major contributor to P. falciparum-iRBC-mediated endothelial barrier disruption. Further supporting these observations, P. falciparum-iRBC-derived factors, such as histones (44, 45), heme (46), and histidine-rich protein II (47), have been implicated in the disruption of endothelial integrity. In patients, an additional mechanism of BBB disruption may be contributing to the pathogenesis of CM, where the binding of specific variants of PfEMP1 to EPCR in brain capillaries can block the receptor constitutive activation through activated protein C and result in increased thrombin activity, which would favor loss of endothelial barrier function (48). Thrombin was shown to enhance the disruptive effect in barrier function of HBMECs by P. falciparum-iRBCs (43), suggesting that this mechanism may contribute to barrier disruption in patients.

Previous work had shown that soluble factors from iRBCs induced apoptosis in human brain vascular endothelial cells (49), and co-culture experiments with lab strains and field isolates of P. falciparum-iRBCs induced apoptosis in endothelial cells (50–52). However, other authors and our own results using primary endothelial cells found low levels of apoptosis induced by iRBCs (43). In our study, neither P. falciparum-iRBCLs nor TNF-α at a physiological concentration in CM patients (1 ng/mL) (53) induced the appearance of abundant apoptotic or necrotic cell populations. These data suggest that although cell death can contribute to the disruption of endothelial barrier integrity, the effects of iRBCLs on HBMEC barrier integrity are not majorly driven by the activation of cell death pathways.

Transcriptional profiling analysis of HBMECs incubated with TNF-α and P. falciparum-iRBCLs confirmed that the distinct stimuli are associated with different gene regulation. Genes upregulated by P. falciparum-iRBCLs and not by TNF-α are enriched for ER stress/unfolded protein response pathways. The ER stress response is largely triggered by the accumulation of unfolded proteins in the ER and is meant to restore ER homeostasis (54, 55), but the prolonged exposure to ER stress can trigger apoptosis by ATF4 induction of the pro-apoptotic transcription factor CHOP (54), which we found uniquely upregulated by P. falciparum-iRBCLs. Although these are pro-apoptotic genes, P. falciparum-iRBCLs also uniquely upregulate DEGs proposed to be anti-apoptotic, like *BDNF* (56), *HMOX1* (57), *HERPUD1* (58), and *HYOU1* (59). Since iRBCLs do not induce apoptosis in HBMECs, these results suggest that anti-apoptotic signals dominate the live/dead HBMEC response. These unique transcriptional signatures further highlight the differences induced by TNF-α and P. falciparum in HBMECs and further support the hypothesis that they affect distinct processes during CM.

Nonetheless, there is also a group of genes that are upregulated by both TNF-α and P. falciparum-iRBCLs, and these are enriched for pathways associated with inflammation. This agrees with previous microarray analysis that found P. falciparum-iRBCs induced the upregulation of inflammatory genes in HBMECs (60, 61). However, our analysis at the protein level reveals that, although transcriptionally upregulated by both TNF-α and P. falciparum iRBCLs, some cytokine and chemokines (IL-6, IL-8, and CCL2) were only secreted by HBMECs stimulated with TNF-α, but not with P. falciparum-iRBCLs. This difference was not a result of defective secretion from HBMECs, but reflected decreased levels of translation of these factors. Similarly, we did not detect increases in surface protein levels of ICAM-1 and VCAM-1 in HBMECs stimulated with P. falciparum-iRBCLs, but the corresponding genes were transcriptionally upregulated. Since our data indicate that both TNF-α and P. falciparum-iRBCLs induce similar increases in general protein translation in HBMECs, the differences observed for protein levels of these immune mediators are probably a result of post-transcriptional mechanisms involved in immune regulation.

Post-transcriptional control of cytokines has extensively been studied and is mainly regulated by the interplay of RNA binding proteins and nucleases that affect mRNA stability (37, 62). The high levels of mRNA but low protein levels of inflammatory cytokines suggest that P. falciparum-iRBCLs may be dampening the inflammatory response in HBMECs. For example, we observed that *ZFP36*, a gene uniquely upregulated by P. falciparum-iRBCLs, encodes an RNA binding protein that decreases the expression of IL-6 and CCL2 in aortic endothelial cells (63). Similarly, we observed a transcriptional increase of *CASP12*, encoding caspase 12, which was shown to suppress immune responses in a mouse malaria model of infection (64).

Analysis of genes involved in the regulation of endothelial barrier integrity identified pathways that promote the disruption of interendothelial junctions. Some of these pathways were upregulated, suggesting a possible direct negative effect of P. falciparum-iRBCLs on barrier integrity, such as the VEGF pathway. However, we also observed other pathways promoting disruption that were downregulated, such as RhoA and FAK, which may suggest a compensatory response trying to mitigate the induced disruption of interendothelial junctions. Further experiments are required to define the role of these pathways in P. falciparum-induced loss of endothelial barrier integrity.

The findings presented here have important implications for the understanding of CM pathogenesis and contribute to validate the hypothesis that CM is a multifactorial complication. Based on our results, we propose that TNF-α is a major driver of the inflammatory processes associated with CM, inducing endothelial activation. On the other hand, P. falciparum would be the key factor inducing the disruption of the BBB. Although part of the transcriptional profile induced by P. falciparum in HBMECs has an inflammatory signature, post-transcriptional control of inflammatory mediators may be dampening this signal, limiting the contribution of P. falciparum to endothelial activation during CM. Since death by CM is linked to brain swelling (3), which is caused preferentially by vasogenic edema in children (4, 65), it is critical to develop therapies targeted at strengthening the BBB. Our findings suggest that prevention of endothelial barrier disruption induced by P. falciparum-iRBCs may be a therapeutic target for CM.

## MATERIALS AND METHODS

### Cell culture.

HBMECs were immortalized as previously described (7). HBMECs were grown in endothelial cell basal medium (ECM) (Sciencell) supplemented with 5% fetal bovine serum (FBS) (Sciencell), 1% endothelial cell growth factor supplement (ECGS) (Sciencell), and 1% penicillin-streptomycin solution (PS) (Sciencell). They were incubated at 37°C in 5% CO_2_. HBMECs were seeded for experiments until they reached 90% to 95% confluence. All experimental conditions were performed in ECM supplemented with only 1% penicillin-streptomycin solution.

### P. falciparum culture, isolation, and lysate preparation.

P. falciparum 3D7 parasites were maintained in erythrocytes (Interstate Blood Bank, Memphis, TN) at 5% hematocrit in RPMI 1640 (Corning) supplemented with 25 mM HEPES (Fisher Scientific), 25 mM sodium bicarbonate (Sigma), 0.5 mM hypoxanthine (Sigma), 0.5% Albumax II (Gibco), and 10 μg/mL gentamicin (Gibco) at 37°C in a gas mixture of 5% O_2_, 5% CO_2_, and 90% N_2_. Parasite cultures were synchronized using 5% sorbitol (Sigma). P. falciparum-infected red blood cells (iRBCs) at the schizont stage were isolated from highly synchronous cultures using magnetic columns (LD MACS separation columns; Miltenyi Biotec). Lysates of uninfected red blood cells (RBCLs) and *P. falciparum-*iRBCs (iRBCLs) were generated by 10 freeze-thaw cycles using liquid nitrogen and a 37°C water bath.

### Calculations of iRBC density in the assays.

The density of iRBCs incubated with over the HBMEC monolayers ranged from 1 × 10^6^ to 8 × 10^6^ iRBCs/cm^2^. We considered that the accumulation of iRBCs in brain capillaries of patients with cerebral malaria is approximately two densely packed layers of iRBCs (6), the average diameter of a P. falciparum schizont is 5.5 μm (66), and the surface of a well in a 96-well plate is 6.94 mm. Using the “Smaller circles within a larger circle” algorithm (The Engineering Toolbox [https://www.engineeringtoolbox.com/smaller-circles-in-larger-circle-d_1849.html]), we estimated that 4 × 10^6^ and 8 × 10^6^ iRBCs/cm^2^ correspond to one and two layers of iRBCs over the HBMECs, respectively.

### Surface detection of ICAM-1 and VCAM-1.

HBMECs were seeded into 96-well plates (Falcon) at 10,500 cells per well and incubated at 37°C for 24 h. HBMECs were then treated with recombinant human TNF-α (PeproTech 300-01A), IFN-γ (PeproTech 300-02), IL-6 (PeproTech 200-06), IL-1β (PeproTech 200-01B), iRBCs, iRBCLs, RBCs, or RBCLs for 20 to 24 h at 37°C. HBMECs were collected using a 0.25% trypsin solution (Corning 25053CI) and washed once with phosphate-buffered saline (PBS) containing 0.1% bovine serum albumin (BSA) (Sigma-Aldrich) and 0.6% sodium citrate (Sigma-Aldrich), referred to as FACS (fluorescence-activated cell sorter) buffer. HBMECs were incubated with phycoerythrin (PE)-conjugated anti-human CD54 (ICAM-1) (Biolegend) and allophycocyanin (APC)-conjugated anti-human CD106 (VCAM-1) (Biolegend) at 1:100 in FACS buffer for 20 min at 4°C. Cells were washed and resuspended in FACS buffer. Samples were processed using a BD FACScalibur (BD Biosciences) and analyzed by FlowJo (Treestar).

### Measurement of barrier integrity.

The changes in impedance were monitored on a xCELLigence RTCA DP analyzer (Agilent) located in a 37°C incubator with 5% CO_2_. The impedance measurements are reported as the cell index (CI), which is a self-calibrated value derived from the ratio of measured impedances. HBMECs were seeded on collagen (40 μg/mL) (Sigma-Aldrich C3867)-coated RTCA PET E-Plate VIEW 16 plates (Agilent) at 15,000 cells per well. HBMECs were incubated for 24 h, in which the state of cellular confluence was measured every 6 h. The last measurement of the 24-h growth curve was used to normalize all CI values after treatment. The effects of all treatments were monitored for 24 h with measurements taken every 15 min.

For xCELLigence experiments involving losartan (Sigma-Aldrich PHR1602), E-plates were prepared and HBMECs were grown to confluence as described above. HBMECs were preincubated with losartan for 1 h with 15-min-interval CI measurements. After the preincubation, RBCLs and iRBCLs were added and changes in CI were monitored for 24 h with 15-min-interval measurements. CI values for these experiments were normalized with the last measurement of the losartan preincubation.

For permeability assays, HBMECs were seeded in inserts (0.4-μm membrane pores) of Transwell plates (Corning 3470) at 15,000 cells per well for 24 h at 37°C. Following treatment, cells were washed and then incubated with 1 mg/mL of fluorescein isothiocyanate (FITC)-dextran (MW of 70,000) (Invitrogen) for 20 min at room temperature. Fluorescence was measured on a Victor X3 plate reader (Perkin Elmer) with excitation at 485 nm and emission at 535 nm.

### Detection of apoptosis.

HBMECs were seeded at 10,500 cells per well in 96-well plates (Falcon) for 24 h at 37°C. After each time point with the stimulus, HBMECs were collected using a 0.25% trypsin solution (Corning 25053CI) and washed once with cold PBS. HBMECs were stained with APC-annexin V (Biolegend) and propidium iodide (5 μg/mL) (BD Biosciences) prepared in 1× annexin V binding buffer (BD Biosciences) for 15 min at room temperature. Samples were immediately processed using a BD FACScalibur (BD Biosciences) and analyzed by FlowJo (Treestar).

### Detection of necrotic cells by immunofluorescence microscopy.

HBMECs were seeded in collagen (40 μg/mL) Sigma-Aldrich C3867)-coated glass bottom 96-well plates (Greiner Bio-One 655981) at 10,500 cells per well for 24 h at 37°C. HBMECs were incubated with medium, RBCLs, iRBCL, and H_2_O_2_ (Fisher Scientific H325) for 3 h and 6 h at 37°C. Following treatment, HBMECs were washed once with endothelial cell basal medium free of phenol red (ECM-PRF) (Sciencell 1001-b-prf). HBMECs were incubated with 10 μg/mL of propidium iodide (BD Biosciences 556463) in ECM-PRF for 15 min at room temperature. HBMECs were washed twice with PBS and fixed with 4% paraformaldehyde (Santa Cruz Biotechnology 281692) for 10 min at room temperature. Following two washes with PBS, HBMECs were permeabilized with 0.25% Triton X-100 (Sigma-Aldrich) in PBS for 10 min at room temperature. HBMECs were washed three times with PBS and then stained with 1× Alexa Fluor 488-phalloidin (Invitrogen A12379) in PBS for 15 min at room temperature. Images were immediately acquired with an Olympus IX70 inverted microscope using a 60× oil objective (Olympus) and processed with MetaMorph Advanced program (Molecular Devices).

The nuclear propidium iodide fluorescence intensity of images was determined using ImageJ (National Institutes of Health) and with a method previously described for quantification of “fluorescence intensity” (https://kpif.umbc.edu/image-processing-resources/imagej-fiji/determining-fluorescence-intensity-and-positive-signal/). HBMECs with a signal 3× the standard deviation (SD) of the untreated (medium) sample were considered positive for propidium iodide staining. The relative frequency was determined by dividing the number of positive cells in the treatment by the total number of positive cells across all treatments.

Following quantification of the propidium iodide fluorescence intensity, HBMECs were stained with a 1:10,000 dilution of Hoechst 33342 (Invitrogen H3570) in water for 5 min at room temperature. HBMECs were then washed three times with PBS. Images were acquired with an Olympus IX70 inverted microscope using a 10× objective (Olympus) and processed with MetaMorph Advanced program (Molecular Devices). The number of nuclei in each image (field) was determined manually.

### RNA sequencing.

HBMECs were seeded in collagen (40 μg/mL) (Sigma-Aldrich C3867)-coated RTCA PET E-Plate VIEW 16 plates at 15,000 cells per well for 24 h at 37°C. HBMECs were then incubated for 9 h in ECM supplemented with 0.5% FBS, 0.1% ECGS, and 0.1% PS. This was followed by a 6 h of incubation with medium, TNF-α (PeproTech), RBCLs, or iRBCLs. Total RNA was extracted with the RNeasy Plus microkit (Qiagen) as per the manufacturer’s instructions. Library preparations were conducted at the NYU Genome Technology Center with poly(A) selection of transcripts. Libraries were sequenced on the NovaSeq 6000 (Illumina) with 100 cycles per sample.

### RNA analysis.

Libraries were sequenced on the Illumina Novaseq. For the bioinformatic pipeline of RNA-seq data, the Seq-N-Slide workflow (source code available at: https://github.com/igordot/sns) was employed. Raw paired-end reads were trimmed via Trimmomatic v0.36 (67), and quality control was performed by using FastQC v0.11.7 (68) and fastQ-Screen v0.13.0 (69). Trimmed reads were mapped to GRCh38/hg38 via STAR v2.7.3a (70). We used subread v1.6.3 (71) to generate the RNA count table. The R package DESeq2 was used for the differential expression analysis (72). The significant differentially expressed genes were determined with the cutoff of log_2_ fold change of >1 or <−1 and a false-discovery rate (FDR) of <0.05. The pathway analysis was performed by using R package goseq (53). We used the principal-component analysis (PCA) and Z-score heat map to visualize DEGs. the visualization was performed by using R packages ggplot2 (73), ComplexHeatmap (74), and ggVennDiagram (75).

### Quantitative real-time PCR.

HBMECs were seeded in 96-well plates (Falcon) at 10,500 cells per well for 24 h at 37°C. HBMECs were incubated with medium, TNF-α (PeproTech), RBCLs, or iRBCLs for 3 and 6 h. For each time point, cell pellets were collected and stored at −80°C until RNA extraction. Total RNA was extracted with the RNeasy Plus microkit (Qiagen) as per the manufacturer’s instructions. Equal amounts of total RNA were reverse transcribed with SuperScript III reverse transcriptase (Invitrogen) using random hexamers (Promega) according to the manufacturer’s protocol. The resulting cDNA was used for the quantitative PCR (qPCR) template. Quantitative real-time PCR was performed on the QuantStudio 7 Flex system (Applied Biosystems) using Power SYBR green (Applied Biosystems). Previously published primer sequences for the *CCL2*, *CXCL8* (76), *IL6*, and *GAPDH* (glyceraldehyde-3-phosphate dehydrogenase) (77) genes were used. Each sample was normalized to *GAPDH* threshold cycle values (*C_T_*), and expression was calculated by the ΔΔ*C_T_* method.

### Cytokine and chemokine measurements.

All supernatants and cell lysates were stored at −80°C until cytokine and chemokine measurement. HBMEC lysates were generated using M-PER mammalian protein extraction reagent (Thermo Scientific) supplemented with 1× cOmplete protease inhibitor cocktail (Roche). In brief, HBMEC monolayers were incubated with the extraction buffer for 5 min with gentle agitation at room temperature. Cell lysates were collected and centrifuged at 14,000 × *g* for 10 min at 4°C to pellet cellular debris. The total protein concentration of cell lysate samples was determined with Pierce 660-nm protein assay kit (Thermo Scientific). Total protein values were used to normalize the levels of intracellular cytokines and chemokines. Cytokines and chemokine levels were measured with the following cytometric bead arrays (CBAs) (BD Biosciences): the human chemokine kit and human inflammatory cytokine kit. These arrays were acquired with a BD FACScalibur (BD Biosciences) and analyzed with FCAP array software (BD Biosciences) or FlowJo (Treestar).

### Protein synthesis detection.

HBMECs were seeded in collagen (40 μg/m) (Sigma-Aldrich C3867)-coated glass bottom 96-well plates (Greiner Bio-One 655981) at 10,500 cells per well for 24 h at 37°C. HBMEC monolayers were incubated with medium, RBCLs, iRBCLs, TNF-α (PeproTech), or TNF-α with cycloheximide (Sigma-Aldrich C7698) for 6 h and 9 h. After each time point, protein synthesis was detected using the Click-iT homopropargylglycine (HPG)-Alexa Fluor 488 protein synthesis assay kit (Invitrogen) as per the manufacturer’s instructions. Images were acquired with an Olympus IX70 inverted microscope using a 60× oil objective (Olympus) and processed with MetaMorph Advanced program (Molecular Devices).

For the cytometric detection of Click-iT HPG-Alexa Fluor 488 (Invitrogen), HBMECs were seeded in 96-well plates (Falcon) at 10,500 cells per well for 24 h at 37°C. Following the same treatment described above, HBMECs were incubated with HPG as per the manufacturer’s instructions. HBMECs were collected using a 0.25% trypsin solution (Corning 25053CI) and washed once with cold PBS. HBMECs were fixed with 4% paraformaldehyde (Santa Cruz Biotechnology 281692) for 15 min at room temperature and then permeabilized with 0.25% Triton X-100 (Sigma-Aldrich) in PBS for 15 min at room temperature. HBMECs were washed with 3% BSA in PBS and stained as per the manufacturer’s instructions, but a 1:2,000 dilution of Alexa Fluor 488 azide was used for cytometric detection. After a wash with 3% BSA in PBS, cell pellets were resuspended in PBS containing 0.1% BSA and 0.02% sodium azide. Samples were processed with a BD FACScalibur (BD Biosciences) and analyzed with FlowJo (Treestar).

### Statistical analysis.

Statistical analyses were performed on Prism 9 (GraphPad Software). The Shapiro-Wilk test was used to test whether the data followed normal distribution. If the data were not normally distributed, the following nonparametric tests were used: the Friedman test (paired data) or the Kruskal-Wallis test (unpaired data) with Dunn’s multiple-comparison test. For all normally distributed data, an unpaired *t* test for comparisons of two groups or one-way analysis of variance (ANOVA) with Tukey’s multiple-comparison test was used. The exact statistical tests used are detailed in the figure legends, and the levels of significance are denoted with asterisks: *, *P* < 0.05; **, *P* < 0.01; ***, *P* < 0.001; ****, *P* < 0.0001.

### Data availability.

The RNAseq data (fastq and count files) reported in this paper have been deposited in the Gene Expression Omnibus (GEO) database (accession no. GSE211439).

## References

[1] WHO. 2021. World malaria report 2021. https://www.who.int/publications/i/item/9789240040496.

[2] Idro R, Marsh K, John CC, Newton CR. 2010. Cerebral malaria: mechanisms of brain injury and strategies for improved neurocognitive outcome. Pediatr Res 68:267–274. doi:10.1203/PDR.0b013e3181eee738. 20606600 PMC3056312

[3] Seydel KB, Kampondeni SD, Valim C, Potchen MJ, Milner DA, Muwalo FW, Birbeck GL, Bradley WG, Fox LL, Glover SJ, Hammond CA, Heyderman RS, Chilingulo CA, Molyneux ME, Taylor TE. 2015. Brain swelling and death in children with cerebral malaria. N Engl J Med 372:1126–1137. doi:10.1056/NEJMoa1400116. 25785970 PMC4450675

[4] Mohanty S, Benjamin LA, Majhi M, Panda P, Kampondeni S, Sahu PK, Mohanty A, Mahanta KC, Pattnaik R, Mohanty RR, Joshi S, Mohanty A, Turnbull IW, Dondorp AM, Taylor TE, Wassmer SC. 2017. Magnetic resonance imaging of cerebral malaria patients reveals distinct pathogenetic processes in different parts of the brain. mSphere 2:e00193-17. doi:10.1128/mSphere.00193-17. 28596990 PMC5463026

[5] Storm J, Jespersen JS, Seydel KB, Szestak T, Mbewe M, Chisala NV, Phula P, Wang CW, Taylor TE, Moxon CA, Lavstsen T, Craig AG. 2019. Cerebral malaria is associated with differential cytoadherence to brain endothelial cells. EMBO Mol Med 11:e9164. doi:10.15252/emmm.201809164. 30610112 PMC6365927

[6] Milner DA, Whitten RO, Kamiza S, Carr R, Liomba G, Dzamalala C, Seydel KB, Molyneux ME, Taylor TE. 2014. The systemic pathology of cerebral malaria in African children. Front Cell Infect Microbiol 4:104. doi:10.3389/fcimb.2014.00104. 25191643 PMC4139913

[7] Gallego-Delgado J, Basu-Roy U, Ty M, Alique M, Fernandez-Arias C, Movila A, Gomes P, Weinstock A, Xu W, Edagha I, Wassmer SC, Walther T, Ruiz-Ortega M, Rodriguez A. 2016. Angiotensin receptors and beta-catenin regulate brain endothelial integrity in malaria. J Clin Invest 126:4016–4029. doi:10.1172/JCI87306. 27643439 PMC5096829

[8] Tripathi AK, Sullivan DJ, Stins MF. 2007. Plasmodium falciparum-infected erythrocytes decrease the integrity of human blood-brain barrier endothelial cell monolayers. J Infect Dis 195:942–950. doi:10.1086/512083. 17330783

[9] Mandala WL, Msefula CL, Gondwe EN, Drayson MT, Molyneux ME, MacLennan CA. 2017. Cytokine profiles in Malawian children presenting with uncomplicated malaria, severe malarial anemia, and cerebral malaria. Clin Vaccine Immunol 24:e00533-16. doi:10.1128/CVI.00533-16. 28122790 PMC5382826

[10] Prakash D, Fesel C, Jain R, Cazenave PA, Mishra GC, Pied S. 2006. Clusters of cytokines determine malaria severity in Plasmodium falciparum-infected patients from endemic areas of central India. J Infect Dis 194:198–207. doi:10.1086/504720. 16779726

[11] Dieye Y, Mbengue B, Dagamajalu S, Fall MM, Loke MF, Nguer CM, Thiam A, Vadivelu J, Dieye A. 2016. Cytokine response during non-cerebral and cerebral malaria: evidence of a failure to control inflammation as a cause of death in African adults. PeerJ 4:e1965. doi:10.7717/peerj.1965. 27168977 PMC4860323

[12] Clark IA, Alleva LM, Budd AC, Cowden WB. 2008. Understanding the role of inflammatory cytokines in malaria and related diseases. Travel Med Infect Dis 6:67–81. doi:10.1016/j.tmaid.2007.07.002. 18342278

[13] O'Carroll SJ, Kho DT, Wiltshire R, Nelson V, Rotimi O, Johnson R, Angel CE, Graham ES. 2015. Pro-inflammatory TNFalpha and IL-1beta differentially regulate the inflammatory phenotype of brain microvascular endothelial cells. J Neuroinflammation 12:131. doi:10.1186/s12974-015-0346-0. 26152369 PMC4506411

[14] Newton CR, Krishna S. 1998. Severe falciparum malaria in children: current understanding of pathophysiology and supportive treatment. Pharmacol Ther 79:1–53. doi:10.1016/S0163-7258(98)00008-4. 9719344

[15] Storm J, Craig AG. 2014. Pathogenesis of cerebral malaria–inflammation and cytoadherence. Front Cell Infect Microbiol 4:100. doi:10.3389/fcimb.2014.00100. 25120958 PMC4114466

[16] Lopez-Ramirez MA, Fischer R, Torres-Badillo CC, Davies HA, Logan K, Pfizenmaier K, Male DK, Sharrack B, Romero IA. 2012. Role of caspases in cytokine-induced barrier breakdown in human brain endothelial cells. J Immunol 189:3130–3139. doi:10.4049/jimmunol.1103460. 22896632

[17] Petrache I, Birukova A, Ramirez SI, Garcia JG, Verin AD. 2003. The role of the microtubules in tumor necrosis factor-alpha-induced endothelial cell permeability. Am J Respir Cell Mol Biol 28:574–581. doi:10.1165/rcmb.2002-0075OC. 12707013

[18] van der Wijk AE, Vogels IMC, van Noorden CJF, Klaassen I, Schlingemann RO. 2017. TNFalpha-induced disruption of the blood-retinal barrier in vitro is regulated by intracellular 3′,5′-cyclic adenosine monophosphate levels. Invest Ophthalmol Vis Sci 58:3496–3505. doi:10.1167/iovs.16-21091. 28715583

[19] Harawa V, Njie M, Kessler A, Choko A, Kumwenda B, Kampondeni S, Potchen M, Kim K, Jaworowski A, Taylor T, Mandala W, Seydel K, Rogerson S. 2018. Brain swelling is independent of peripheral plasma cytokine levels in Malawian children with cerebral malaria. Malar J 17:435. doi:10.1186/s12936-018-2590-0. 30477519 PMC6260579

[20] Pappa V, Seydel K, Gupta S, Feintuch CM, Potchen MJ, Kampondeni S, Goldman-Yassen A, Veenstra M, Lopez L, Kim RS, Berman JW, Taylor T, Daily JP. 2015. Lipid metabolites of the phospholipase A2 pathway and inflammatory cytokines are associated with brain volume in paediatric cerebral malaria. Malar J 14:513. doi:10.1186/s12936-015-1036-1. 26691993 PMC4687364

[21] Riggle BA, Miller LH, Pierce SK. 2020. Desperately seeking therapies for cerebral malaria. J Immunol 204:327–334. doi:10.4049/jimmunol.1900829. 31907275 PMC6951433

[22] Varo R, Crowley VM, Sitoe A, Madrid L, Serghides L, Kain KC, Bassat Q. 2018. Adjunctive therapy for severe malaria: a review and critical appraisal. Malar J 17:47. doi:10.1186/s12936-018-2195-7. 29361945 PMC5781278

[23] Lell B, Köhler C, Wamola B, Olola CH, Kivaya E, Kokwaro G, Wypij D, Mithwani S, Taylor TE, Kremsner PG, Newton CRJC. 2010. Pentoxifylline as an adjunct therapy in children with cerebral malaria. Malar J 9:368. doi:10.1186/1475-2875-9-368. 21176151 PMC3152769

[24] van Hensbroek MB, Palmer A, Onyiorah E, Schneider G, Jaffar S, Dolan G, Memming H, Frenkel J, Enwere G, Bennett S, Kwiatkowski D, Greenwood B. 1996. The effect of a monoclonal antibody to tumor necrosis factor on survival from childhood cerebral malaria. J Infect Dis 174:1091–1097. doi:10.1093/infdis/174.5.1091. 8896514

[25] Glennon EKK, Dankwa S, Smith JD, Kaushansky A. 2018. Opportunities for host-targeted therapies for malaria. Trends Parasitol 34:843–860. doi:10.1016/j.pt.2018.07.011. 30122551 PMC6168423

[26] Pober JS, Sessa WC. 2007. Evolving functions of endothelial cells in inflammation. Nat Rev Immunol 7:803–815. doi:10.1038/nri2171. 17893694

[27] Gallego-Delgado J, Rodriguez A. 2017. Rupture and release: a role for soluble erythrocyte content in the pathology of cerebral malaria. Trends Parasitol 33:832–835. doi:10.1016/j.pt.2017.06.005. 28709836 PMC5685654

[28] Rochfort KD, Collins LE, McLoughlin A, Cummins PM. 2016. Tumour necrosis factor-alpha-mediated disruption of cerebrovascular endothelial barrier integrity in vitro involves the production of proinflammatory interleukin-6. J Neurochem 136:564–572. doi:10.1111/jnc.13408. 26499872

[29] Rastogi S, Rizwani W, Joshi B, Kunigal S, Chellappan SP. 2012. TNF-alpha response of vascular endothelial and vascular smooth muscle cells involve differential utilization of ASK1 kinase and p73. Cell Death Differ 19:274–283. doi:10.1038/cdd.2011.93. 21738216 PMC3191254

[30] Shi JH, Sun SC. 2018. Tumor necrosis factor receptor-associated factor regulation of nuclear factor kappaB and mitogen-activated protein kinase pathways. Front Immunol 9:1849. doi:10.3389/fimmu.2018.01849. 30140268 PMC6094638

[31] Honda K, Taniguchi T. 2006. IRFs: master regulators of signalling by Toll-like receptors and cytosolic pattern-recognition receptors. Nat Rev Immunol 6:644–658. doi:10.1038/nri1900. 16932750

[32] Lee J, Borboa AK, Chun HB, Baird A, Eliceiri BP. 2010. Conditional deletion of the focal adhesion kinase FAK alters remodeling of the blood-brain barrier in glioma. Cancer Res 70:10131–10140. doi:10.1158/0008-5472.CAN-10-2740. 21159635 PMC3059220

[33] Mayhan WG. 1999. VEGF increases permeability of the blood-brain barrier via a nitric oxide synthase/cGMP-dependent pathway. Am J Physiol 276:C1148–C1153. doi:10.1152/ajpcell.1999.276.5.C1148. 10329964

[34] Spindler V, Schlegel N, Waschke J. 2010. Role of GTPases in control of microvascular permeability. Cardiovasc Res 87:243–253. doi:10.1093/cvr/cvq086. 20299335

[35] Götz A, Tang MS, Ty MC, Arama C, Ongoiba A, Doumtabe D, Traore B, Crompton PD, Loke P, Rodriguez A. 2017. Atypical activation of dendritic cells by Plasmodium falciparum. Proc Natl Acad Sci USA 114:E10568–E10577. doi:10.1073/pnas.1708383114. 29162686 PMC5724257

[36] Mita-Mendoza NK, Magallon-Tejada A, Parmar P, Furtado R, Aldrich M, Saidi A, Taylor T, Smith J, Seydel K, Daily JP. 2020. Dimethyl fumarate reduces TNF and Plasmodium falciparum induced brain endothelium activation in vitro. Malar J 19:376. doi:10.1186/s12936-020-03447-7. 33087130 PMC7579885

[37] Anderson P. 2008. Post-transcriptional control of cytokine production. Nat Immunol 9:353–359. doi:10.1038/ni1584. 18349815

[38] Sanwlani R, Gangoda L. 2021. Role of extracellular vesicles in cell death and inflammation. Cells 10:2663. doi:10.3390/cells10102663. 34685643 PMC8534608

[39] Eisenhut M. 2015. The evidence for a role of vasospasm in the pathogenesis of cerebral malaria. Malar J 14:405. doi:10.1186/s12936-015-0928-4. 26463364 PMC4603731

[40] Brown H, Hien TT, Day N, Mai NT, Chuong LV, Chau TT, Loc PP, Phu NH, Bethell D, Farrar J, Gatter K, White N, Turner G. 1999. Evidence of blood-brain barrier dysfunction in human cerebral malaria. Neuropathol Appl Neurobiol 25:331–340. doi:10.1046/j.1365-2990.1999.00188.x. 10476050

[41] Moxon CA, Chisala NV, Wassmer SC, Taylor TE, Seydel KB, Molyneux ME, Faragher B, Kennedy N, Toh C-H, Craig AG, Heyderman RS. 2014. Persistent endothelial activation and inflammation after Plasmodium falciparum infection in Malawian children. J Infect Dis 209:610–615. doi:10.1093/infdis/jit419. 24048963 PMC3903368

[42] Newton CR, Taylor TE, Whitten RO. 1998. Pathophysiology of fatal falciparum malaria in African children. Am J Trop Med Hyg 58:673–683. doi:10.4269/ajtmh.1998.58.673. 9598460

[43] Avril M, Benjamin M, Dols MM, Smith JD. 2019. Interplay of Plasmodium falciparum and thrombin in brain endothelial barrier disruption. Sci Rep 9:13142. doi:10.1038/s41598-019-49530-1. 31511575 PMC6739390

[44] Gillrie MR, Lee K, Gowda DC, Davis SP, Monestier M, Cui L, Hien TT, Day NPJ, Ho M. 2012. Plasmodium falciparum histones induce endothelial proinflammatory response and barrier dysfunction. Am J Pathol 180:1028–1039. doi:10.1016/j.ajpath.2011.11.037. 22260922 PMC3448071

[45] Moxon C, Alhamdi Y, Storm J, Toh J, Ko JY, Murphy G, Taylor T, Seydel K, Wang G, García-Car G, Molyneux M, Craig A, Abrams S, Toh C-H. 2020. Parasite histones mediate blood-brain barrier disruption in cerebral malaria. Clin Med (Lond) 20:s96–s7. doi:10.7861/clinmed.20-2-s96. 32409404 PMC7243569

[46] Liu M, Dickinson-Copeland C, Hassana S, Stiles JK. 2016. Plasmodium-infected erythrocytes (pRBC) induce endothelial cell apoptosis via a heme-mediated signaling pathway. Drug Des Devel Ther 10:1009–1018. doi:10.2147/DDDT.S96863. PMC4780719 27042002

[47] Pal P, Daniels BP, Oskman A, Diamond MS, Klein RS, Goldberg DE. 2016. Plasmodium falciparum histidine-rich protein II compromises brain endothelial barriers and may promote cerebral malaria pathogenesis. mBio 7:e00617-16. doi:10.1128/mBio.00617-16. 27273825 PMC4959673

[48] Bernabeu M, Smith JD. 2017. EPCR and malaria severity: the center of a perfect storm. Trends Parasitol 33:295–308. doi:10.1016/j.pt.2016.11.004. 27939609 PMC5376506

[49] Wilson NO, Huang M-B, Anderson W, Bond V, Powell M, Thompson WE, Armah HB, Adjei AA, Gyasi R, Tettey Y, Stiles JK. 2008. Soluble factors from Plasmodium falciparum-infected erythrocytes induce apoptosis in human brain vascular endothelial and neuroglia cells. Mol Biochem Parasitol 162:172–176. doi:10.1016/j.molbiopara.2008.09.003. 18848585 PMC2671222

[50] Essone JCBB, N'Dilimabaka N, Ondzaga J, Lekana-Douki JB, Mba DN, Deloron P, Mazier D, Gay F, Touré Ndouo FS. 2017. Comparison of apoptosis in human primary pulmonary endothelial cells and a brain microvascular endothelial cell line co-cultured with Plasmodium falciparum field isolates. BMC Infect Dis 17:454. doi:10.1186/s12879-017-2552-0. 28655315 PMC5488356

[51] Pino P, Vouldoukis I, Kolb JP, Mahmoudi N, Desportes-Livage I, Bricaire F, Danis M, Dugas B, Mazier D. 2003. Plasmodium falciparum-infected erythrocyte adhesion induces caspase activation and apoptosis in human endothelial cells. J Infect Dis 187:1283–1290. doi:10.1086/373992. 12696008

[52] Touré FS, Ouwe-Missi-Oukem-Boyer O, Bisvigou U, Moussa O, Rogier C, Pino P, Mazier D, Bisser S. 2008. Apoptosis: a potential triggering mechanism of neurological manifestation in Plasmodium falciparum malaria. Parasite Immunol 30:47–51. doi:10.1111/j.1365-3024.2007.00986.x. 18086016

[53] Young MD, Wakefield MJ, Smyth GK, Oshlack A. 2010. Gene Ontology analysis for RNA-seq: accounting for selection bias. Genome Biol 11:R14. doi:10.1186/gb-2010-11-2-r14. 20132535 PMC2872874

[54] Hetz C. 2012. The unfolded protein response: controlling cell fate decisions under ER stress and beyond. Nat Rev Mol Cell Biol 13:89–102. doi:10.1038/nrm3270. 22251901

[55] Rutkowski DT, Hegde RS. 2010. Regulation of basal cellular physiology by the homeostatic unfolded protein response. J Cell Biol 189:783–794. doi:10.1083/jcb.201003138. 20513765 PMC2878945

[56] Almeida RD, Manadas BJ, Melo CV, Gomes JR, Mendes CS, Grãos MM, Carvalho RF, Carvalho AP, Duarte CB. 2005. Neuroprotection by BDNF against glutamate-induced apoptotic cell death is mediated by ERK and PI3-kinase pathways. Cell Death Differ 12:1329–1343. doi:10.1038/sj.cdd.4401662. 15905876

[57] Brouard S, Otterbein LE, Anrather J, Tobiasch E, Bach FH, Choi AM, Soares MP. 2000. Carbon monoxide generated by heme oxygenase 1 suppresses endothelial cell apoptosis. J Exp Med 192:1015–1026. doi:10.1084/jem.192.7.1015. 11015442 PMC2193315

[58] Paredes F, Parra V, Torrealba N, Navarro-Marquez M, Gatica D, Bravo-Sagua R, Troncoso R, Pennanen C, Quiroga C, Chiong M, Caesar C, Taylor WR, Molgó J, San Martin A, Jaimovich E, Lavandero S. 2016. HERPUD1 protects against oxidative stress-induced apoptosis through downregulation of the inositol 1,4,5-trisphosphate receptor. Free Radic Biol Med 90:206–218. doi:10.1016/j.freeradbiomed.2015.11.024. 26616647 PMC4710961

[59] Rao S, Oyang L, Liang J, Yi P, Han Y, Luo X, Xia L, Lin J, Tan S, Hu J, Wang H, Tang L, Pan Q, Tang Y, Zhou Y, Liao Q. 2021. Biological function of HYOU1 in tumors and other diseases. Onco Targets Ther 14:1727–1735. doi:10.2147/OTT.S297332. 33707955 PMC7943547

[60] Tripathi AK, Sha W, Shulaev V, Stins MF, Sullivan DJ, Jr. 2009. Plasmodium falciparum-infected erythrocytes induce NF-kappaB regulated inflammatory pathways in human cerebral endothelium. Blood 114:4243–4252. doi:10.1182/blood-2009-06-226415. 19713460 PMC2925626

[61] Chakravorty SJ, Carret C, Nash GB, Ivens A, Szestak T, Craig AG. 2007. Altered phenotype and gene transcription in endothelial cells, induced by Plasmodium falciparum-infected red blood cells: pathogenic or protective? Int J Parasitol 37:975–987. doi:10.1016/j.ijpara.2007.02.006. 17383656 PMC1906861

[62] Vlasova-St Louis I, Bohjanen PR. 2017. Post-transcriptional regulation of cytokine and growth factor signaling in cancer. Cytokine Growth Factor Rev 33:83–93. doi:10.1016/j.cytogfr.2016.11.004. 27956133 PMC5337147

[63] Zhang H, Taylor WR, Joseph G, Caracciolo V, Gonzales DM, Sidell N, Seli E, Blackshear PJ, Kallen CB. 2013. mRNA-binding protein ZFP36 is expressed in atherosclerotic lesions and reduces inflammation in aortic endothelial cells. Arterioscler Thromb Vasc Biol 33:1212–1220. doi:10.1161/ATVBAHA.113.301496. 23559629 PMC3844532

[64] Labbé K, Miu J, Yeretssian G, Serghides L, Tam M, Finney CA, Erdman LK, Goulet M-L, Kain KC, Stevenson MM, Saleh M. 2010. Caspase-12 dampens the immune response to malaria independently of the inflammasome by targeting NF-kappaB signaling. J Immunol 185:5495–5502. doi:10.4049/jimmunol.1002517. 20876354

[65] Sahu PK, Duffy FJ, Dankwa S, Vishnyakova M, Majhi M, Pirpamer L, Vigdorovich V, Bage J, Maharana S, Mandala W, Rogerson SJ, Seydel KB, Taylor TE, Kim K, Sather DN, Mohanty A, Mohanty RR, Mohanty A, Pattnaik R, Aitchison JD, Hoffman A, Mohanty S, Smith JD, Bernabeu M, Wassmer SC. 2021. Determinants of brain swelling in pediatric and adult cerebral malaria. JCI Insight 6:e145823. doi:10.1172/jci.insight.145823. 34549725 PMC8492338

[66] Esposito A, Choimet J-B, Skepper JN, Mauritz JMA, Lew VL, Kaminski CF, Tiffert T. 2010. Quantitative imaging of human red blood cells infected with Plasmodium falciparum. Biophys J 99:953–960. doi:10.1016/j.bpj.2010.04.065. 20682274 PMC2913174

[67] Bolger AM, Lohse M, Usadel B. 2014. Trimmomatic: a flexible trimmer for Illumina sequence data. Bioinformatics 30:2114–2120. doi:10.1093/bioinformatics/btu170. 24695404 PMC4103590

[68] Andrews S. 2010. FASTQC. A quality control tool for high throughput sequence data. https://www.bioinformatics.babraham.ac.uk/projects/fastqc/.

[69] Wingett SW, Andrews S. 2018. FastQ Screen: a tool for multi-genome mapping and quality control. F1000Res 7:1338. doi:10.12688/f1000research.15931.2. 30254741 PMC6124377

[70] Dobin A, Davis CA, Schlesinger F, Drenkow J, Zaleski C, Jha S, Batut P, Chaisson M, Gingeras TR. 2013. STAR: ultrafast universal RNA-seq aligner. Bioinformatics 29:15–21. doi:10.1093/bioinformatics/bts635. 23104886 PMC3530905

[71] Liao Y, Smyth GK, Shi W. 2014. featureCounts: an efficient general purpose program for assigning sequence reads to genomic features. Bioinformatics 30:923–930. doi:10.1093/bioinformatics/btt656. 24227677

[72] Love MI, Huber W, Anders S. 2014. Moderated estimation of fold change and dispersion for RNA-seq data with DESeq2. Genome Biol 15:550. doi:10.1186/s13059-014-0550-8. 25516281 PMC4302049

[73] Wickham H. 2016. ggplot2: elegant graphics for data analysis: Springer-Verlag, New York, NY.

[74] Gu Z, Eils R, Schlesner M. 2016. Complex heatmaps reveal patterns and correlations in multidimensional genomic data. Bioinformatics 32:2847–2849. doi:10.1093/bioinformatics/btw313. 27207943

[75] Gao CH, Yu G, Dusa A. 2021. ggVennDiagram: a 'ggplot2' implement of Venn Diagram. https://github.com/gaospecial/ggVennDiagram.

[76] Jimenez-Munguia I, Tomeckova Z, Mochnacova E, Bhide K, Majerova P, Bhide M. 2021. Transcriptomic analysis of human brain microvascular endothelial cells exposed to laminin binding protein (adhesion lipoprotein) and Streptococcus pneumoniae. Sci Rep 11:7970. doi:10.1038/s41598-021-87021-4. 33846455 PMC8041795

[77] Sato A, Kamekura R, Kawata K, Kawada M, Jitsukawa S, Yamashita K, Sato N, Himi T, Ichimiya S. 2016. Novel mechanisms of compromised lymphatic endothelial cell homeostasis in obesity: the role of leptin in lymphatic endothelial cell tube formation and proliferation. PLoS One 11:e0158408. doi:10.1371/journal.pone.0158408. 27366905 PMC4930203

