# Supplementary figures and images for: Plasmodium falciparum and TNF-α Differentially Regulate Inflammatory and Barrier Integrity Pathways in Human Brain Endothelial Cells
Source: mBio. 2022 Aug 29;13(5):e01746-22. doi: 10.1128/mbio.01746-22 (PMC9601155; doi:10.1128/mbio.01746-22)

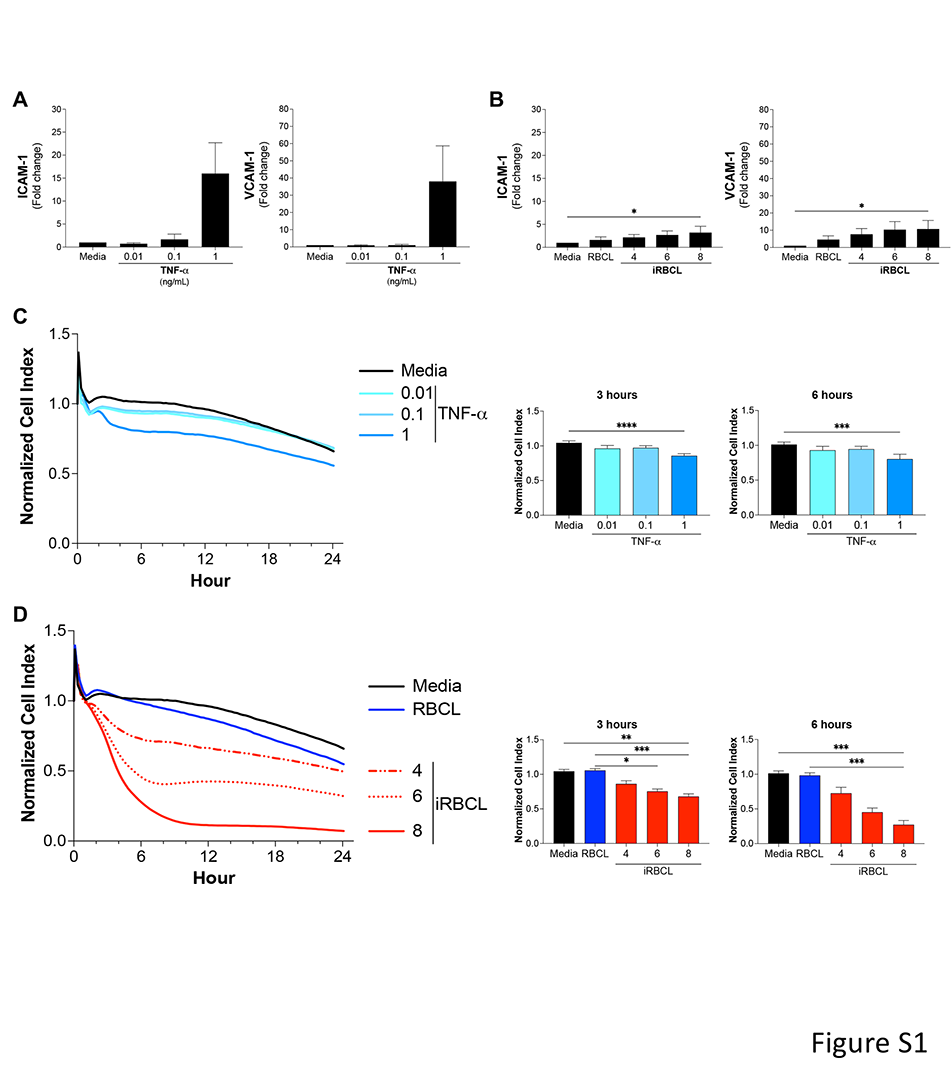

Supplement: FIG S1 [file mbio.01746-22-s0004.tif]

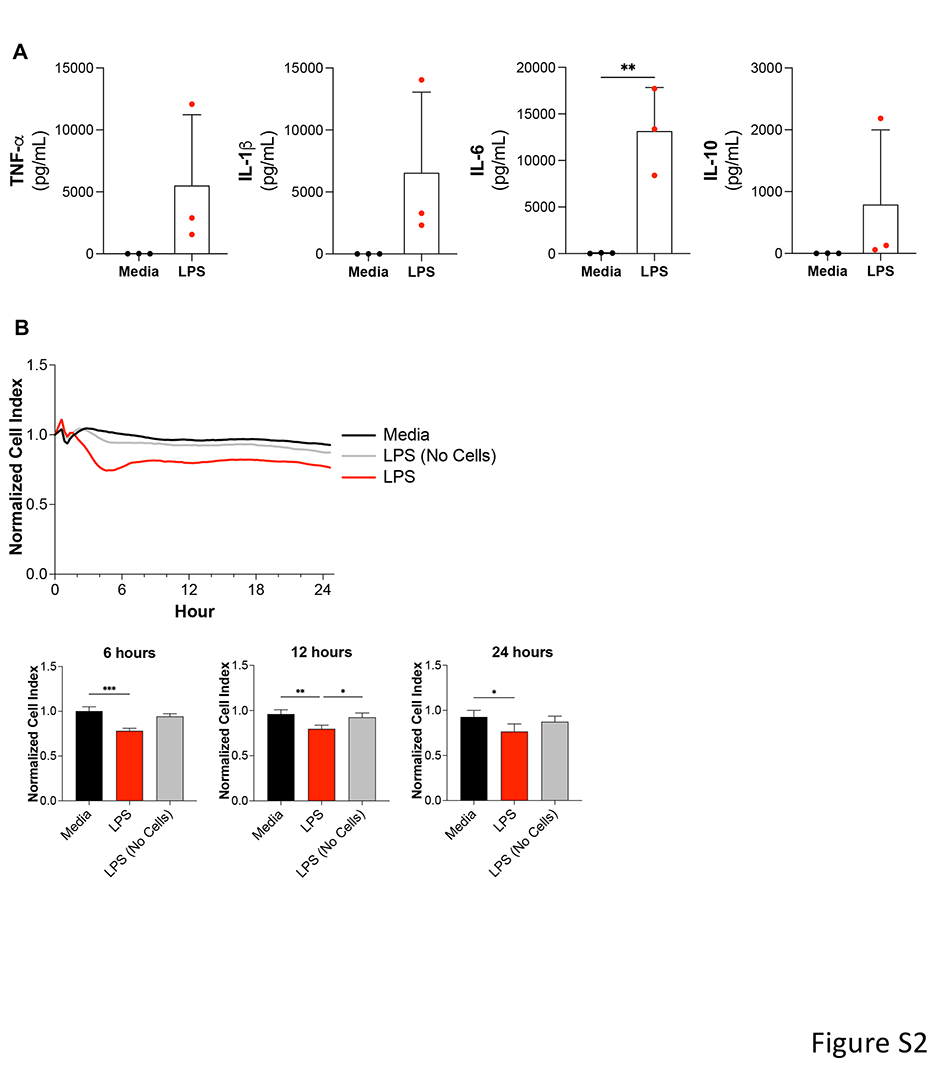

Supplement: FIG S2 [file mbio.01746-22-s0005.tif]

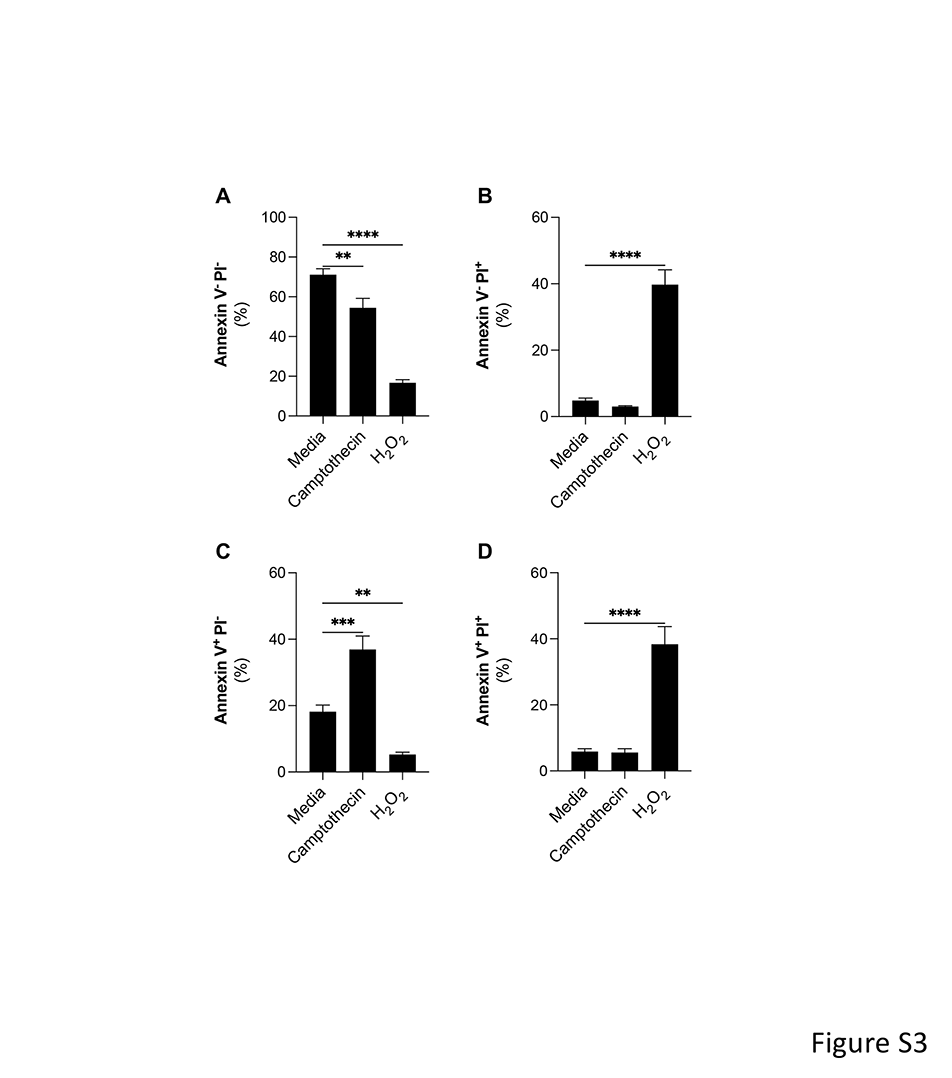

Supplement: FIG S3 [file mbio.01746-22-s0006.tif]

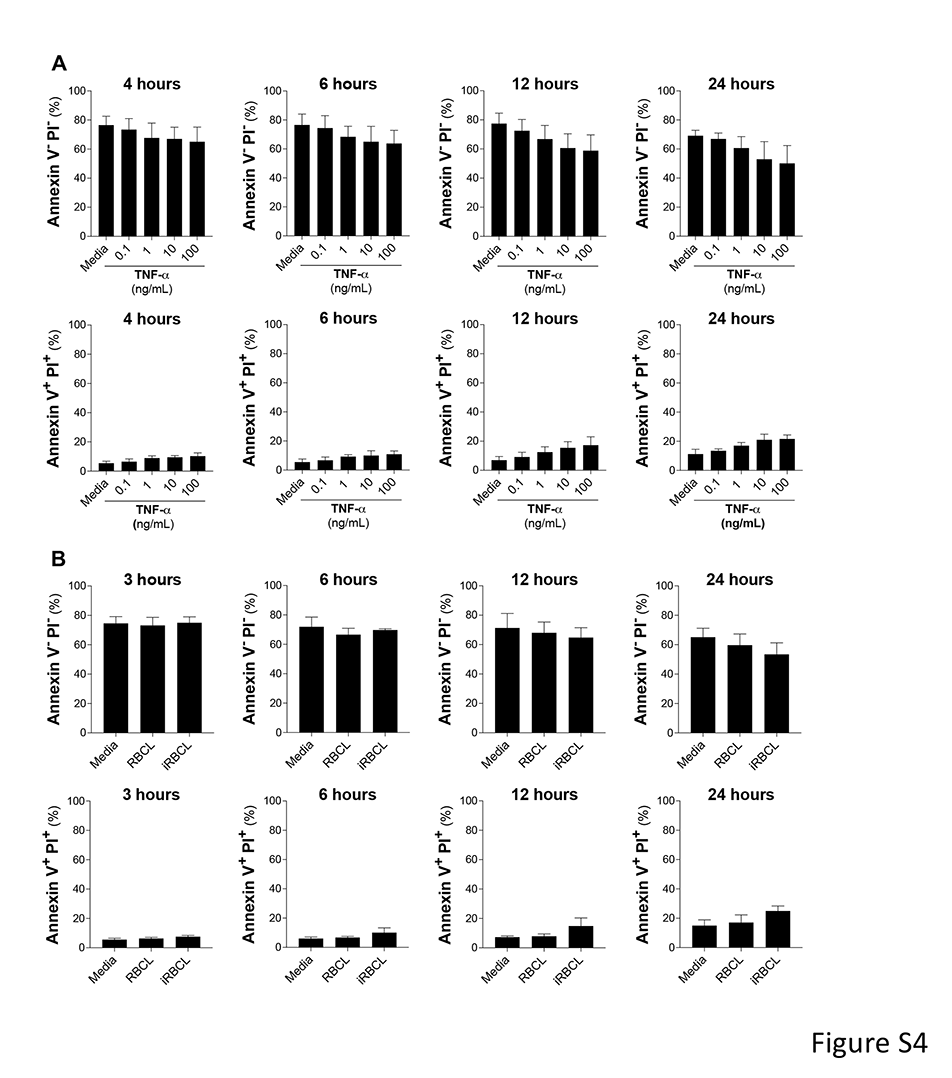

Supplement: FIG S4 [file mbio.01746-22-s0007.tif]

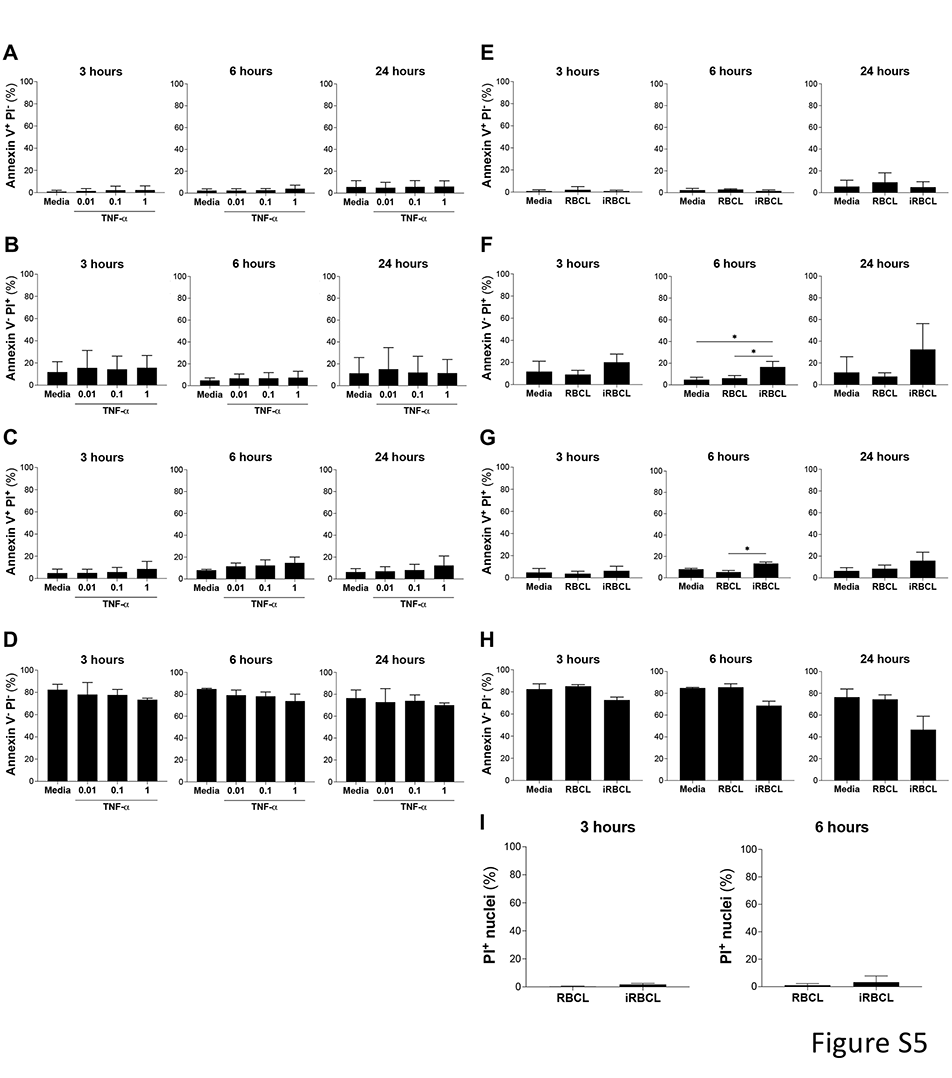

Supplement: FIG S5 [file mbio.01746-22-s0008.tif]

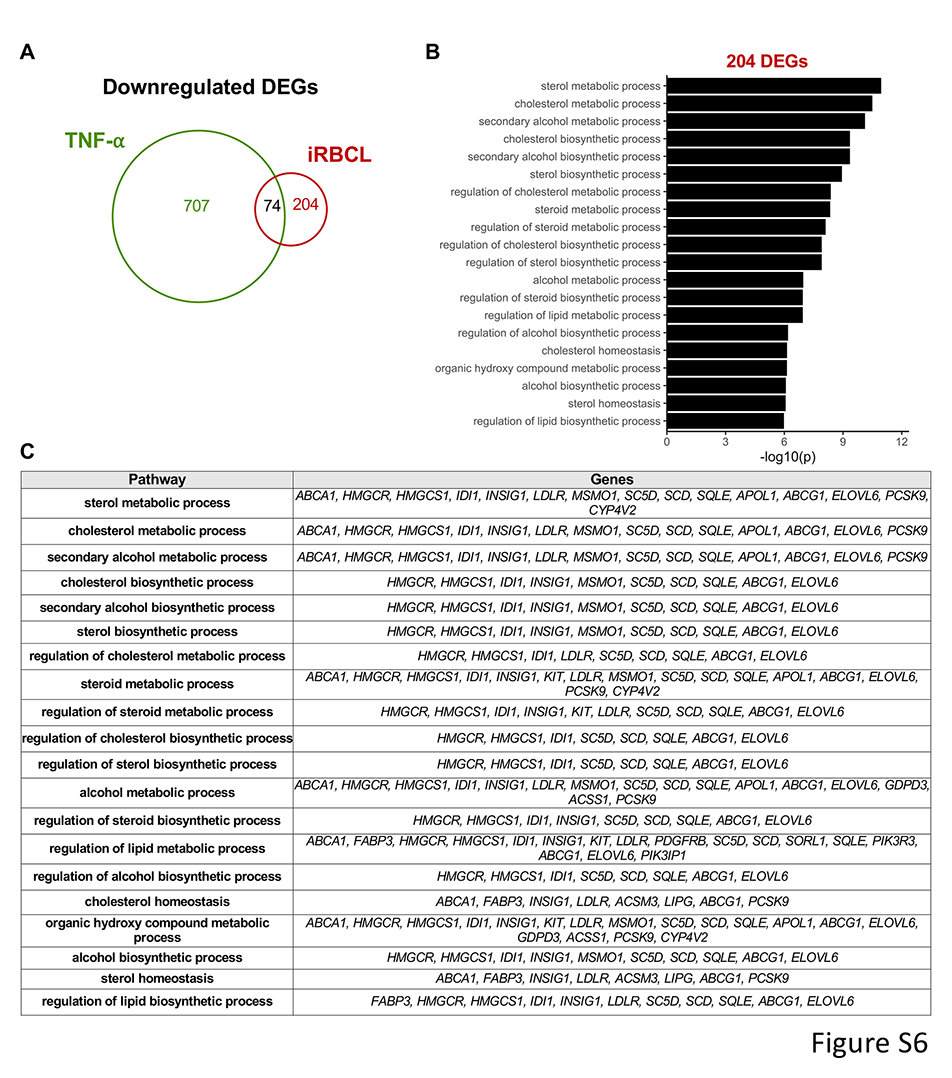

Supplement: FIG S6 [file mbio.01746-22-s0009.tif]

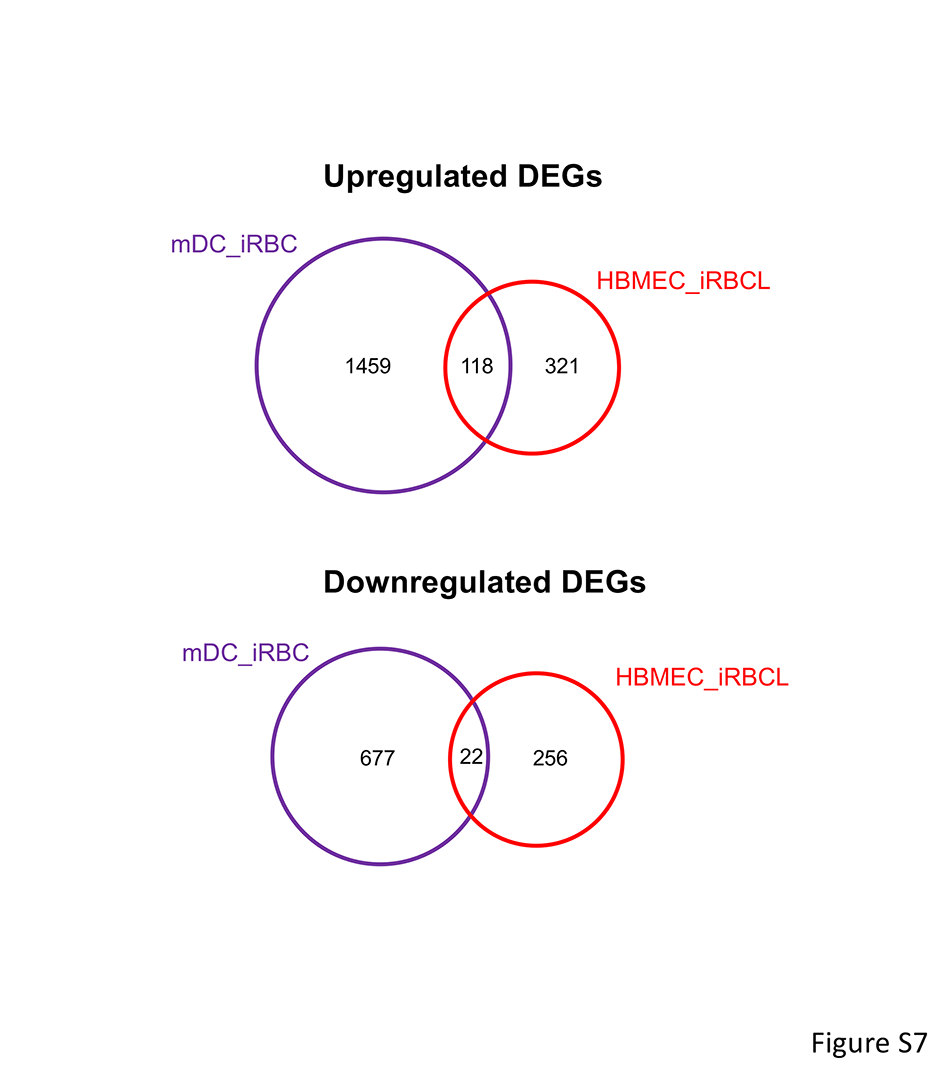

Supplement: FIG S7 [file mbio.01746-22-s0010.tif]
