# Supplementary material for: Plasmodium falciparum and TNF-α Differentially Regulate Inflammatory and Barrier Integrity Pathways in Human Brain Endothelial Cells
Source: mBio. 2022 Aug 29;13(5):e01746-22. doi: 10.1128/mbio.01746-22 (PMC9601155; doi:10.1128/mbio.01746-22)
Supplement: TABLE S1 [file mbio.01746-22-s0001.docx]

**Table S1. Comparison of cell death vs loss of barrier integrity by TNF-𝛂 and iRBCL in HBMEC.**

|  |  | **Increase in cell death* (%)** | **Decrease in Cell index^#^ (%)** | **Ratio^$^** |
| --- | --- | --- | --- | --- |
| **TNF**-𝛂 **(100ng/ml)** | 4h | 11 | 26.3 | 2.4 |
|  | 6h | 13 | 25.4 | 2.0 |
| **iRBCL** | 3h | -2 | 18.9 | -9.4 |
|  | 6h | 3.4 | 56.7 | 16.6 |

^*^Cell death was calculated as the addition of the % of necrotic, early apoptotic and late apoptotic cells.

^*,#^Data are calculated relative to controls: media and RBCL, for TNF-𝛂 and iRBCL, respectively. From Figs. 2A, 3B, 4A and S4

^#^Decrease in cell index is proportional to the loss of barrier integrity

^$^Decrease in cell index (%) / Increase in cell death (%)
