# Supplementary material for: Plasmodium falciparum and TNF-α Differentially Regulate Inflammatory and Barrier Integrity Pathways in Human Brain Endothelial Cells
Source: mBio. 2022 Aug 29;13(5):e01746-22. doi: 10.1128/mbio.01746-22 (PMC9601155; doi:10.1128/mbio.01746-22)
Supplement: Table S2 [file mbio.01746-22-s0002.docx]

**Table S2A. Gene lists of top 20 pathways mapped by DEGs uniquely upregulated by TNF-𝛂 in HBMEC.**

| **Pathway** | **Genes** |
| --- | --- |
| **response to type I interferon** | *ADAR, BST2, IFI6, GBP2, HLA-A, HLA-B, HLA-C, HLA-E, HLA-F, HLA-G, HLA-H, IFI27, IFI35, IFIT2, IFIT1, IFIT3, IFNAR2, IFNB1, IRF1, IRF2, IRF5, IRF7, ISG20, MX1, MX2, MYD88, OAS1, OAS2, OAS3, PSMB8, SP100, STAT1, STAT2, IFITM1, OASL, ISG15, IKBKE, IFITM3, USP18, SAMHD1, XAF1, SHFL, NLRC5, RSAD2* |
| **type I interferon signaling pathway** | *ADAR, BST2, IFI6, GBP2, HLA-A, HLA-B, HLA-C, HLA-E, HLA-F, HLA-G, HLA-H, IFI27, IFI35 IFIT2, IFIT1, IFIT3, IFNAR2, IFNB1, IRF1, IRF2, IRF5, IRF7, ISG20, MX1, MX2, MYD88, OAS1, OAS2, OAS3, PSMB8, SP100, STAT1, STAT2, IFITM1, OASL, ISG15, IKBKE, IFITM3, USP18, SAMHD1, XAF1, NLRC5, RSAD2* |
| **cellular response to type I interferon** | *ADAR, BST2, IFI6, GBP2, HLA-A, HLA-B, HLA-C, HLA-E, HLA-F, HLA-G, HLA-H, IFI27, IFI35, IFIT2, IFIT1, IFIT3, IFNAR2, IFNB1, IRF1, IRF2, IRF5, IRF7, ISG20, MX1, MX2, MYD88, OAS1, OAS2, OAS3, PSMB8, SP100, STAT1, STAT2, IFITM1, OASL, ISG15, IKBKE, IFITM3, USP18, SAMHD1, XAF1, NLRC5, RSAD2* |
| **defense response to virus** | *ADAR, BIRC2, BST2, CD40, F2RL1, IFI6, GBP1, GBP3, IFI27, IFIT2, IFIT1, IFIT3, IFNAR2, IFNB1, IL12RB1, IL15, CXCL10, IRF1, IRF2, IRF5, IRF7, ISG20, CXCL9, MX1, MX2, OAS1, OAS2, OAS3, PLSCR1, PML, EIF2AK2, STAT1, STAT2, TLR3, TRAF3, IFITM1, OASL, ISG15, TRIM22, IFITM3, IFI44L, DDX58, IFIT5, SAMHD1, CD207, HERC5, SHFL, DDX60, ZC3HAV1, APOBEC3G, RTP4, IFIH1, DHX58, UNC93B1, PARP9, NLRC5, TRIM5, RSAD2, SLFN11, APOBEC3D, TICAM1, DTX3L, APOBEC3F* |
| **response to virus** | *ADAR, BIRC2, BST2, CD40, CDK6, F2RL1, IFI6, GBP1, GBP3, IFI27, IFIT2, IFIT1, IFIT3, IFNAR2, IFNB1, IL12RB1, IL15, CXCL10, IRF1, IRF2, IRF5, IRF7, ISG20, LGALS9, CXCL9, MX1, MX2, OAS1, OAS2, OAS3, PLSCR1, PML, EIF2AK2, CCL5, STAT1, STAT2, TLR3, TRAF3, TNFSF4, IFITM1, OASL, ISG15, IKBKE, TRIM22, IFITM3, IFI44, IFI44L, DDX58, IFIT5, SAMHD1, CD207, HERC5, SHFL, BATF3, DDX60, ZC3HAV1, APOBEC3G, RTP4, IFIH1, DHX58, UNC93B1, PARP9, NLRC5, TRIM5, RSAD2, SLFN11, APOBEC3D, TICAM1, DTX3L, APOBEC3F* |
| **response to interferon-gamma** | *BST2, CASP1, CD40, CD47, CYP27B1, GBP1, GBP2, GBP3, GCH1, HLA-A, HLA-B, HLA-C, HLA-E, HLA-F, HLA-G, HLA-H, IL12RB1, IRF1, IRF2, IRF5, IRF7, JAK2, LGALS9, CIITA, MT2A, OAS1, OAS2, OAS3, PML, PTAFR, CCL5, CCL7, CCL17, SP100, TRIM21, STAT1, TLR2, TLR3, TRIM25, IFITM1, OASL, SOCS1, NMI, TRIM22, IFITM3, IFI30, NUB1, PARP14, SHFL, CXCL16, PARP9, NLRC5, TRIM5, GBP4, GBP5* |
| **cellular response to interferon-gamma** | *CASP1, CD47, GBP1, GBP2, GBP3, HLA-A, HLA-B, HLA-C, HLA-E, HLA-F, HLA-G, HLA-H, IL12RB1, IRF1, IRF2, IRF5, IRF7, JAK2, LGALS9, CIITA, MT2A, OAS1, OAS2, OAS3, PML, PTAFR, CCL5, CCL7, CCL17, SP100, TRIM21, STAT1, TLR2, TLR3, TRIM25, OASL, SOCS1, NMI, TRIM22, IFI30, PARP14, PARP9, NLRC5, TRIM5, GBP4, GBP5* |
| **interferon-gamma-mediated signaling pathway** | *GBP1, GBP2, HLA-A, HLA-B, HLA-C, HLA-E, HLA-F, HLA-G, HLA-H, IRF1, IRF2, IRF5, IRF7, JAK2, CIITA, MT2A, OAS1, OAS2, OAS3, PML, PTAFR, SP100, TRIM21, STAT1, TRIM25, OASL, SOCS1, NMI, TRIM22, IFI30, PARP14, PARP9, NLRC5, TRIM5* |
| **regulation of defense response** | *ADAM8, ADAR, ADORA2A, BIRC2, SERPING1, CASP1, CD47, CYLD, EGFR, F2RL1, HLA-A, HLA-B, HLA-E, HLA-F, HLA-G, IFIT1, IFNAR2, IFNB1, IL1R1, IL12RB1, IL15, IDO1, IRF1, IRF7, JAK2, LAG3, LGALS9, LTA, SMAD3, MUC1, MUC5AC, MYD88, SERPINE1, PLSCR1, PSMB8, PSMB9, PSMB10, PSME1, PSME2, NECTIN2, CCL5, SLC7A2, STAT1, TLR2, TLR3, TRAF3, TNFSF4, SEMA7A, SOCS1, SPHK1, NMI, IKBKE, OPTN, TNIP1, TRAFD1, DUSP10, USP18, MGLL, SBNO2, DDX58, SAMHD1, HERC5, GHRL, PARP14, DDX60, APOBEC3G, NOD2, NFKBIZ, DHX58, PARP9, NLRC5, TRIM5, GBP5, SH2D1B, PIK3AP1, LACC1, TICAM1, DTX3L, APOBEC3F* |
| **regulation of cytokine production** | *ADAM8, BIRC2, ARRB1, BST2, CASP1, CD40, CD47, CD74, CYLD, F2RL1, ACKR1, LRRC32, GBP1, HLA-A, HLA-B, HLA-E, HLA-F, HLA-G, HTR2B, IFNB1, IL1R1, IL12RB1, IL15, IDO1, IRF1, IRF5, IRF7, ITGB8, JAK2, JAK3, LGALS9, LTA, SMAD3, MYD88, SERPINE1, MAP2K3, EIF2AK2, PTAFR, TRIM21, STAT1, STAT6, TGFB3, TLR2, TLR3, TRAF2, TRAF3, TNFSF4, TNFRSF4, TRIM25, FZD5, SEMA7A, SOCS1, IL18R1, SPHK1, NMI, UBE2L6, IL27RA, LITAF, ISG15, IKBKE, BTN3A3, TRIM16, BTN3A1, DTX4, DDX58, PANX1, PRKD2, TNFRSF21, DLL1, HERC5, GHRL, DDX60, INAVA, NOD2, IFIH1, DHX58, PDCD1LG2, NLRC5, NAV3, RSAD2, GBP5, TICAM1* |
| **negative regulation of viral life cycle** | *BST2, IFIT1, IFNB1, ISG20, MX1, OAS1, OAS2, OAS3, PLSCR1, PML, EIF2AK2, CCL5, TRIM21, TRIM25, IFITM1, OASL, ISG15, TNIP1, IFITM3, IFIT5, SHFL, ZC3HAV1, APOBEC3G, PARP10, TRIM5, RSAD2, APOBEC3D, APOBEC3F* |
| **regulation of viral life cycle** | *ADAR, BST2, CD74, IFI27, IFIT1, IFNB1, ISG20, LGALS9, MX1, OAS1, OAS2, OAS3, PLSCR1, PML, EIF2AK2, NECTIN2, CCL5, TRIM21, TRIM25, IFITM1, OASL, ISG15, TRIM14, TNIP1, TRIM22, IFITM3, IFIT5, LAMP3, SHFL, ZC3HAV1, APOBEC3G, PARP10, TRIM5, RSAD2, APOBEC3D, APOBEC3F* |
| **negative regulation of viral process** | *BST2, IFIT1, IFNB1, ISG20, MX1, OAS1, OAS2, OAS3, PLSCR1, PML, EIF2AK2, CCL5, TRIM21, STAT1, TRIM25, IFITM1, OASL, ISG15, TRIM14, TNIP1, IFITM3, IFIT5, SHFL, ZC3HAV1, APOBEC3G, PARP10, TRIM5, RSAD2, APOBEC3D, APOBEC3F* |
| **regulation of immune effector process** | *BIRC2, CFB, BST2, SERPING1, C1R, C1S, C4A, CD40, CD47, CD74, CR1L, F2RL1, HLA-A, HLA-B, HLA-E, HLA-F, HLA-G, HLA-H, IFIT1, IFNB1, IL1R1, IL4R, IL7R, IL12RB1, IL15, JAK3, LAG3, LGALS9, LTA, PTAFR, NECTIN2, STAT1, STAT6, TGFB3, TLR3, TRAF2, TRAF3, TNFSF4, TNFRSF4, FZD5, SEMA7A, IL18R1, IL27RA, DUSP10, DDX58, HERC5, DDX60, APOBEC3G, NOD2, NFKBIZ, DHX58, PARP9, RSAD2, SH2D1B, TICAM1, DTX3L, APOBEC3F, APLF* |
| **negative regulation of viral genome replication** | *BST2, IFIT1, IFNB1, ISG20, MX1, OAS1, OAS2, OAS3, PLSCR1, EIF2AK2, CCL5, IFITM1, OASL, ISG15, TNIP1, IFITM3, IFIT5, SHFL, ZC3HAV1, APOBEC3G, PARP10, RSAD2, APOBEC3D, APOBEC3F* |
| **positive regulation of immune response** | *ADAM8, CFB, SERPING1, C1R, C1S, C4A, CD38, CD40, CD47, CD74, CR1L, CYLD, F2RL1, FCGR2A, GBP1, HLA-A, HLA-B, HLA-E, HLA-F, HLA-G, HLA-H, IFNB1, IL1R1, IL4R, IL12RB1, IL15, IDO1, IRF7, LAG3, LGALS9, LTA, MUC1, MUC5AC, PAX5, PLSCR1, PSMB8, PSMB9, PSMB10, PSME1, PSME2, PTAFR, NECTIN2, CCL5, STAT6, TRAF2, TNFSF4, FZD5, SEMA7A, IL18R1, IL27RA, THEMIS2, IKBKE, BTN3A3, RBCK1, TNFSF13B, BTN3A1, DDX58, PRKD2, TNFRSF21, RNF31, NOD2, NFKBIZ, PARP9, NLRC5, TRIM5, RSAD2, GBP5, SH2D1B, TICAM1* |
| **regulation of response to biotic stimulus** | *ADAM8, ADAR, BIRC2, SERPING1, F2RL1, HLA-A, HLA-B, HLA-E, HLA-F, HLA-G, IFIT1, IFNAR2, IFNB1, IL12RB1, IL15, IRF1, IRF7, JAK2, LAG3, LGALS9, MUC1, MUC5AC, PLSCR1, PSMB8, PSMB9, PSMB10, PSME1, PSME2, NECTIN2, CCL5, STAT1, TRAF3, SOCS1, NMI, IKBKE, OPTN, TRAFD1, DUSP10, USP18, DDX58, SAMHD1, HERC5, PARP14, DDX60, APOBEC3G, DHX58, PARP9, NLRC5, TRIM5, GBP5, SH2D1B, DTX3L, APOBEC3F* |
| **positive regulation of response to external stimulus** | *ADAM8, CD47, CD74, CCR4, CSF1, CYP27B1, EGFR, F2RL1, HLA-E, HLA-F, HLA-G, IFNB1, IL15, IDO1, CXCL10, IRF7, JAK2, LAG3, LGALS9, LTA, SMAD3, MUC1, MUC5AC, MYD88, P2RX4, SERPINE1, PLSCR1, LGMN, PSMB8, PSMB9, PSMB10, PSME1, PSME2, NECTIN2, CCL5, CCL7, TLR2, TLR3, TNFSF4, IKBKE, OPTN, TNIP1, DAPK2, PRKD2, GHRL, DDX60, CAMK1D, NOD2, NFKBIZ, DHX58, PARP9, NLRC5, TRIM5, GBP5, SH2D1B, IL34, TICAM1, TUBB2B* |
| **regulation of viral genome replication** | *ADAR, BST2, IFI27, IFIT1, IFNB1, ISG20, MX1, OAS1, OAS2, OAS3, PLSCR1, EIF2AK2, CCL5, IFITM1, OASL, ISG15, TNIP1, IFITM3, IFIT5, SHFL, ZC3HAV1, APOBEC3G, PARP10, RSAD2, APOBEC3D, APOBEC3F* |
| **adaptive immune response** | *PRDM1, SERPING1, C1R, C1S, C4A, CD40, CD70, CD74, CTSS, HLA-A, HLA-B, HLA-C, HLA-E, HLA-F, HLA-G, HLA-H, IFNB1, IL1R1, IL4R, IL7R, IL12RB1, IRF1, IRF7, JAK2, JAK3, LAG3, LTA, NBN, NECTIN2, STAT6, TAP1, TAP2, TRAF2, TNFSF4, FZD5, IL18R1, IL27RA, IL18BP, BTN3A3, TNFSF13B, BTN3A1, DUSP10, PRKD2, LAMP3, TNFRSF21, SAMSN1, NFKBIZ, PDCD1LG2, UNC93B1, RSAD2, SH2D1B, RNF19B, APLF* |

**Table S2B.** **Gene lists of top 20 pathways mapped by DEGs uniquely upregulated by *P. falciparum* iRBCL in HBMEC.**

| **Pathway** | **Genes** |
| --- | --- |
| **nucleosome** | *H2AC8, H2BC5, H4C9, H2AC6, H2BC8, H2BC6, H2BC4, H2BC17, H2BC21, H4C8, H2AC11, H2BC11, H2BC12, H2BU1* |
| **DNA packaging complex** | *H2AC8, H2BC5, H4C9, H2AC6, H2BC8, H2BC6, H2BC4, H2BC17, H2BC21, H4C8, H2AC11, H2BC11, H2BC12, H2BU1* |
| **protein heterodimerization activity** | *ATF4, CEBPB, GADD45A, DDIT3, H2AC8, H2BC5, NR4A1, NR4A2, H4C9, H2AC6, H2BC8, H2BC6, H2BC4, H2BC17, H2BC21, H4C8, DGKD, H2AC11, H2BC11, H2BC12, H2BU1* |
| **protein-DNA complex** | *DDIT3, FOS, H2AC8, H2BC5, H4C9, H2AC6, H2BC8, H2BC6, H2BC4, H2BC17, H2BC21, H4C8, H2AC11, H2BC11, NUPR1, H2BC12, H2BU1* |
| **response to endoplasmic reticulum stress** | *ASNS, ATF4, CEBPB, CTH, DDIT3, HSPA1A, HSPA5, JUN, DNAJB9, MANF, HERPUD1, HYOU1, FICD, PPP1R15A, SDF2L1, NUPR1, TRIB3, CHAC1, SESN2, SYVN1, DERL3* |
| **growth factor activity** | *AREG, BDNF, BMP2, CSF3, HBEGF, NRG1, IL11, INHBA, KITLG, TGFB2, VEGFA, MANF, GDF15, OSGIN1, INHBE* |
| **response to topologically incorrect protein** | *ASNS, ATF4, CTH, DDIT3, HSPA1A, HSPA1B, HSPA5, HSPA6, DNAJB9, MANF, HERPUD1, HYOU1, FICD, PPP1R15A, SDF2L1, CHAC1, SYVN1, DERL3* |
| **response to unfolded protein** | *ASNS, ATF4, CTH, DDIT3, HSPA1A, HSPA1B, HSPA5, HSPA6, DNAJB9, MANF, HERPUD1, HYOU1, FICD, PPP1R15A, CHAC1, SYVN1, DERL3* |
| **cellular response to topologically incorrect protein** | *ASNS, ATF4, CTH, DDIT3, HSPA1A, HSPA1B, HSPA5, HSPA6, DNAJB9, HERPUD1, HYOU1, FICD, PPP1R15A, SDF2L1, SYVN1, DERL3* |
| **regulation of response to endoplasmic reticulum stress** | *DDIT3, HSPA1A, HSPA5, DNAJB9, MANF, HERPUD1, HYOU1, FICD, PPP1R15A, NUPR1, SYVN1, DERL3* |
| **cellular response to unfolded protein** | *ASNS, ATF4, CTH, DDIT3, HSPA1A, HSPA1B, HSPA5, HSPA6, DNAJB9, HERPUD1, HYOU1, FICD, PPP1R15A, SYVN1, DERL3* |
| **intrinsic apoptotic signaling pathway in response to endoplasmic reticulum stress** | *ATF4, CEBPB, DDIT3, HSPA1A, HERPUD1, HYOU1, PPP1R15A, TRIB3, CHAC1, SYVN1* |
| **innate immune response in mucosa** | *H2BC8, H2BC6, H2BC4, H2BC21, H2BC11, H2BC12* |
| **rhythmic process** | *ATF4, BDNF, EGR1, EGR3, HSPA5, INHBA, JUN, KISS1, NFIL3, SIAH2, TEF, TGFB2, KLF10, PER2, NR1D1, NR1D2* |
| **apoptotic signaling pathway** | *ATF4, BDNF, BIK, CEBPB, CTH, DDIT3, HMOX1, HSPA1A, HSPA1B, INHBA, JUN, KITLG, NR4A2, SIAH2, TGFB2, HERPUD1, HYOU1, PPP1R15A, NUPR1, TNFRSF12A, DDIT4, TRIB3, CHAC1, SYVN1, CASP12* |
| **stress response to metal ion** | *HSPA5, MT1E, MT1F, MT1G, MT1X, SLC30A1* |
| **mucosal immune response** | *H2BC8, H2BC6, H2BC4, H2BC21, H2BC11, H2BC12* |
| **amino acid import across plasma membrane** | *SLC1A5, SLC3A2, SLC6A9, SLC7A1, SLC7A5, PER2, SLC7A11* |
| **organ or tissue specific immune response** | *H2BC8, H2BC6, H2BC4, H2BC21, H2BC11, H2BC12* |
| **misfolded protein binding** | *HSPA1A, HSPA1B, HSPA5, HSPA6, DNAJB9, SDF2L1, DERL3* |

**Table S2C.** **Gene lists of top 20 pathways mapped by DEGs upregulated by TNF-𝛂 and *P. falciparum* iRBCL in HBMEC.**

| **Pathway** | **Genes** |
| --- | --- |
| **response to lipopolysaccharide** | *CSF2, CXCL1, CXCL2, CXCL3, ICAM1, IL1A, IL1B, IL6, CXCL8, IRAK2, NFKB1, NFKB2, NFKBIA, NFKBIB, PTGS2, CCL2, CXCL6, CX3CL1, SELE, TNF, TNFAIP3, XBP1, RIPK2, CLDN1, TRIB1, NOCT, CD274, TNIP3, ZC3H12A* |
| **response to molecule of bacterial origin** | *CSF2, CXCL1, CXCL2, CXCL3, ICAM1, IL1A, IL1B, IL6, CXCL8, IRAK2, NFKB1, NFKB2, NFKBIA, NFKBIB, PTGS2, CCL2, CXCL6, CX3CL1, SELE, TNF, TNFAIP3, XBP1, RIPK2, CLDN1, TRIB1, NOCT, CD274, TNIP3, ZC3H12A* |
| **cellular response to lipopolysaccharide** | *CSF2, CXCL1, CXCL2, CXCL3, ICAM1, IL1A, IL1B, IL6, CXCL8, IRAK2, NFKB1, NFKBIA, NFKBIB, CCL2, CXCL6, CX3CL1, TNF, TNFAIP3, XBP1, RIPK2, TRIB1, CD274, TNIP3, ZC3H12A* |
| **inflammatory response** | *BIRC3, BCL6, BDKRB2, C3, ETS1, F3, CXCL1, CXCL2, CXCL3, ICAM1, IL1A, IL1B, IL6, CXCL8, IRAK2, NFKB1, NFKBIA, PTGS2, PTX3, RELB, CCL2, CCL20, CXCL6, CX3CL1, SELE, TNF, TNFAIP3, PLA2G4C, RIPK2, KLF4, HDAC9, IL23A, TNIP3, ZC3H12A, C2CD4A, BCL6B, C2CD4B* |
| **cellular response to molecule of bacterial origin** | *CSF2, CXCL1, CXCL2, CXCL3, ICAM1, IL1A, IL1B, IL6, CXCL8, IRAK2, NFKB1, NFKBIA, NFKBIB, CCL2, CXCL6, CX3CL1, TNF, TNFAIP3, XBP1, RIPK2, TRIB1, CD274, TNIP3, ZC3H12A* |
| **response to bacterium** | *BCL3, C3, CSF2, CXCL1, CXCL2, CXCL3, ICAM1, IL1A, IL1B, IL6, CXCL8, I RAK2, NFKB1, NFKB2, NFKBIA, NFKBIB, PTGS2, CCL2, CCL20, CXCL6, CX3CL1, SELE, TNF, TNFAIP3, XBP1, RIPK2, CLDN1, TRIB1, NOCT, CD274, IL23A, TNIP3, ZC3H12A* |
| **cellular response to biotic stimulus** | *CSF2, CXCL1, CXCL2, CXCL3, ICAM1, IL1A, IL1B, IL6, CXCL8, IRAK2, NFKB1, NFKBIA, NFKBIB, CCL2, CXCL6, CX3CL1, TNF, TNFAIP3, XBP1, RIPK2, TRIB1, CD274, TNIP3, ZC3H12A* |
| **cytokine activity** | *CSF2, CXCL1, CXCL2, CXCL3, IL1A, IL1B, IL6, CXCL8, LIF, LTB, CCL2, CCL20, CXCL6, CX3CL1, TNF, TNFSF18, IL32, TNFSF15, EBI3, IL23A, VSTM1* |
| **receptor regulator activity** | *CSF2, FGF5, CXCL1, CXCL2, CXCL3, IL1A, IL1B, IL6, CXCL8, LIF, LTB, CCL2, CCL20, CXCL6, CX3CL1, TNF, VEGFC, STC2, TNFSF18, IL32, TNFSF15, EBI3, SEMA3A, FST, IL23A, ADM2, VSTM1* |
| **cytokine receptor binding** | *CSF2, CXCL1, CXCL2, CXCL3, IL1A, IL1B, IL6, CXCL8, LIF, LTB, CCL2, CCL20, CXCL6, CX3CL1, TNF, TRAF1, VEGFC, TNFSF18, TNFSF15, EBI3, IL23A* |
| **receptor ligand activity** | *CSF2, FGF5, CXCL1, CXCL2, CXCL3, IL1A, IL1B, IL6, CXCL8, LIF, LTB, CCL2, CCL20, CXCL6, CX3CL1, TNF, VEGFC, STC2, TNFSF18, IL32, TNFSF15, EBI3, SEMA3A, IL23A, ADM2, VSTM1* |
| **signaling receptor activator activity** | *CSF2, FGF5, CXCL1, CXCL2, CXCL3, I L1A, IL1B, IL6, CXCL8, LIF, LTB, CCL2, CCL20, CXCL6, CX3CL1, TNF, VEGFC, STC2, TNFSF18, IL32, TNFSF15, EBI3, SEMA3A, IL23A, ADM2, VSTM1* |
| **regulation of cytokine production** | *BIRC3, BCL3, BCL6, C3, CSF2, F3, IL1A, IL1B, IL6, LTB, NFKB1, NFKB2, POU2F2, PTGS2, RELB, CXCL6, CX3CL1, TNF, TNFAIP3, XBP1, NR4A3, RIPK2, CD83, KLF4, HDAC9, EBI3, CD274, IL23A, ZC3H12A, CGAS, BCL6B* |
| **cellular response to lipid** | *CSF2, CXCL1, CXCL2, CXCL3, ICAM1, IL1A, IL1B, IL6, CXCL8, IRAK2, NFKB1, NFKBIA, NFKBIB, CCL2, CXCL6, CX3CL1, TNF, TNFAIP3, XBP1, NR4A3, NRIP1, RIPK2, CLDN1, KLF4, TRIB1, CD274, TNIP3, ZC3H12A* |
| **positive regulation of cellular component movement** | *BCL6, S1PR1, ETS1, F3, ICAM1, IL1B, IL6, CXCL8, LAMC2, RELN, PTGS2, CCL20, CX3CL1, SELE, SOD2, TNF, VEGFC, XBP1, NR4A3, TNFSF18, CLDN1, HDAC9, SEMA3A, CD274, IL23A, ACKR3, ZC3H12A* |
| **positive regulation of cell migration** | *S1PR1, ETS1, F3, ICAM1, IL1B, IL6, CXCL8, LAMC2, RELN, PTGS2, CCL20, CX3CL1, SELE, SOD2, TNF, VEGFC, XBP1, NR4A3, TNFSF18, CLDN1, HDAC9, SEMA3A, CD274, IL23A, ACKR3, ZC3H12A* |
| **positive regulation of cell motility** | *S1PR1, ETS1, F3, ICAM1, IL1B, IL6, CXCL8, LAMC2, RELN, PTGS2, CCL20, CX3CL1, SELE, SOD2, TNF, VEGFC, XBP1, NR4A3, TNFSF18, CLDN1, HDAC9, SEMA3A, CD274, IL23A, ACKR3, ZC3H12A* |
| **positive regulation of locomotion** | *S1PR1, ETS1, F3, ICAM1, IL1B, IL6, CXCL8, LAMC2, RELN, PTGS2, CCL20, CX3CL1, SELE, SOD2, TNF, VEGFC, XBP1, NR4A3, TNFSF18, CLDN1, HDAC9, SEMA3A, CD274, IL23A, ACKR3, ZC3H12A* |
| **positive regulation of cytokine production** | *BIRC3, BCL3, C3, CSF2, F3, IL1A, IL1B, IL6, LTB, NFKB1, NFKB2, POU2F2, PTGS2, CX3CL1, TNF, XBP1, NR4A3, RIPK2, CD83, EBI3, CD274, IL23A, CGAS* |
| **regulation of cell adhesion** | *BCL6, S1PR1, ETS1, ICAM1, IL1A, IL1B, IL6, CXCL8, LIF, CCL2, CX3CL1, SDC4, SELE, TNF, VCAM1, XBP1, NR4A3, RIPK2, TNFSF18, CD83, KLF4,*  *EBI3, RND1, CD274, IL23A, ZC3H12A, C2CD4A, C2CD4B* |
