# Supplementary material for: Plasmodium falciparum and TNF-α Differentially Regulate Inflammatory and Barrier Integrity Pathways in Human Brain Endothelial Cells
Source: mBio. 2022 Aug 29;13(5):e01746-22. doi: 10.1128/mbio.01746-22 (PMC9601155; doi:10.1128/mbio.01746-22)
Supplement: TABLE S3 [file mbio.01746-22-s0003.docx]

**Table S3.** **List of inter-endothelial junction-related pathways regulated by *P. falciparum* iRBCL in HBMEC.**

| **Pathway (Ingenuity Pathway Analysis)** | **z-score** | **Interpretation** | **Genes** |
| --- | --- | --- | --- |
| Tumor Microenvironment Pathway | 1.134 | Upregulated | *CD274, COL1A2, FGF5, JUN, PIK3R3, VEGFA, VEGFC* |
| ILK Signaling | 0.447 | Upregulated | *ARHGEF6, JUN, MYO18B, PIK3R3, RND1, VEGFA, VEGFC* |
| FAK Signaling | -1.155 | Downregulated | *ADRA1D, ARHGEF6, BDKRB2, COL1A2, ITGA6, JUN, LGR4, LPAR1, LPAR2, PCDH12, PDGFRB, PIK3R3* |
| Hepatic Fibrosis Signaling Pathway | 0 | - | *COL1A2, ITGA6, JUN, PDGFRB, PIK3R3, RND1, VEGFA, VEGFC, WNT9A* |
| Breast Cancer Regulation by Stathmin1 | 0.632 | Upregulated | *ADRA1D, ARHGEF6, BDKRB2, JUN, LGR4, LPAR1, LPAR2, PIK3R3, VEGFA, VEGFC* |
| Wound Healing Signaling Pathway | 0.378 | Upregulated | *COL1A2, ITGA6, JUN, LAMB3, LAMB2, VEGFA, VEGFC* |
| eNOS Signaling | 0.816 | Upregulated | *BDKRB2, LPAR1, LPAR2, PIK3R3, VEGFA, VEGFC* |
| Role of Tissue Factor in Cancer | - | - | *FGF5, ITGA6, PIK3R3, VEGFA, VEGFC* |
| RHOA Signaling | -0.447 | Downregulated | *ARHGAP9, CDC42EP1, DLC1, LPAR1, LPAR2* |
| PCP (Planar Cell Polarity) Pathway | 0 | - | *JUN, LGR4, RSPO3, WNT9A* |
| MSP-RON Signaling in Cancer Cells | 1.342 | Upregulated | *ITGA6, JUN, PIK3R3, VEGFA, VEGFC* |
| Phagosome Formation | 0.333 | Upregulated | *ADRA1D, BDKRB2, ITGA6, LGR4, LPAR1, LPAR2, MYO18B, PIK3R3, PLA2G4C* |
| Colorectal Cancer Metastasis Signaling | 0.447 | Upregulated | *JUN, PIK3R3, RND1, VEGFA, VEGFC, WNT9A* |
| Signaling by Rho Family GTPases | 0 | - | *ARHGEF6, CDC42EP1, ITGA6, JUN, PIK3R3, RND1* |
| VEGF Family Ligand-Receptor Interactions | 1 | Upregulated | *PIK3R3, PLA2G4C, VEGFA, VEGFC* |
| CREB Signaling in Neurons | 0 | - | *ADRA1D, BDKRB2, LGR4, LPAR1, LPAR2, PDGFRB, PIK3R3, VEGFA* |
| Hepatic Fibrosis | - | - | *COL1A2, MYO18B, PDGFRB, VEGFA, VEGFC* |
| Pulmonary Fibrosis Idiopathic Signaling Pathway | -0.816 | Downregulated | *COL1A2, JUN, LPAR1, PDGFRB, PIK3R3, WNT9A* |
| IL-8 Signaling | 1 | Upregulated | *JUN, PIK3R3, RND1, VEGFA, VEGFC* |
| RHOGDI Signaling | - | - | *ARHGAP9, ARHGEF6, DLC1, ITGA6, RND1* |
| Axonal Guidance Signaling | - | - | *ARHGEF6, ITGA6, PIK3R3, RND1, VEGFA, VEGFC, WNT9A* |
| Nitric Oxide Signaling in the Cardiovascular System | 1 | Upregulated | *BDKRB2, PIK3R3, VEGFA, VEGFC* |
| PAK Signaling | - | - | *ARHGEF6, ITGA6, PDGFRB, PIK3R3* |
| GP6 Signaling Pathway | 0 | - | *COL1A2, LAMB3, LAMC2, PIK3R3* |
| Actin Cytoskeleton Signaling | - | - | *ARHGEF6, FGF5, ITGA6, MYO18B, PIK3R3* |
| Gα12/13 Signaling | 0 | - | *JUN, LPAR1, LPAR2, PIK3R3* |
| Reelin Signaling in Neurons | - | - | *ARHGEF6, ARHGEF37, PIK3R3, RELN* |
| Ovarian Cancer Signaling | - | - | *PIK3R3, VEGFA, VEGFC, WNT9A* |
| Molecular Mechanisms of Cancer | - | - | *ARHGEF6, ITGA6, JUN, PIK3R3, RND1, WNT9A* |
| Glioblastoma Multiforme Signaling | - | - | *PDGFRB, PIK3R3, RND1, WNT9A* |
